# Piperazine linked chitosan schiff base nanoparticles as a novel antibiofilm and antibacterial strategy against clinically relevant pathogens

**DOI:** 10.1038/s41598-026-63745-z

**Published:** 2026-08-06

**Authors:** Mona M. Soliman, Abdelrahman Essam Bayoumy, Amira A. Hamed, Refaat M. Gabre, Mohamed A. Abd-Elhakeem, Ismail A. Abdelhamid, Ahmed M. Elgamal

**Affiliations:** 1 Department of Botany and Microbiology, Faculty of Science, Cairo University, P.O. 12613, Giza, Egypt; 2 Department of Biology, Basic Science Center, Misr University for Science and Technology (MUST), Giza, Egypt; 3 Department of Chemistry, Faculty of Science, Cairo University, P.O. 12613, Giza, Egypt; 4 Department of Biotechnology, Faculty of Science, Cairo University, P.O. 12613, Giza, Egypt; 5 Department of pharmaceutical biotechnology- college of biotechnology- MISR university for science and technology, Giza, Egypt

**Keywords:** Antimicrobial activity, Antibiofilm, Chitosan nanoparticles, Molecular docking, Minimum inhibitory concentration (MIC), Biotechnology, Drug discovery, Microbiology

## Abstract

The development of multifunctional antimicrobial materials capable of targeting both planktonic bacteria and biofilm-associated infections remains a critical challenge in combating antimicrobial resistance. In this study, a novel piperazine-linked chitosan Schiff base (Cs-TPA-PiP) and its ionically crosslinked nanoparticle formulation (Cs-TPA-PiP NPs) were synthesized and structurally characterized. The antimicrobial potential of both Cs-TPA-PiP and Cs-TPA-PiP NPs was evaluated against a panel of nine standard clinically significant bacterial strains. The compounds demonstrated significant and broad-spectrum antibacterial activity. The minimum inhibitory concentration (MIC) values demonstrated potent efficacy, with Cs-TPA-PiP and its Cs-TPA-PiP NPs ranging from 0.63 to 2.50 mg/mL and 1.00–5.00 mg/mL, respectively. Notably, both agents exhibited a strong dose-dependent inhibitory effect on biofilm formation. While Cs-TPA-PiP showed lower MIC values against planktonic cells, the corresponding Cs-TPA-PiP NPs with an ultra-small spherical size of 15.6 nm exhibited superior antibiofilm performance, ranging from 73.00% to 95.00% inhibition of biofilm biomass at 1× MIC in strong biofilm-producing strains. Transmission electron microscopy (TEM) confirmed severe morphological alterations and membrane disruption in treated bacterial cells, consistent with a membrane-targeting mechanism. In silico molecular docking studies suggested that the compound has favorable binding affinity for the critical bacterial cell wall target, Sortase A, thereby identifying it as a potential theoretical target requiring further validation. Our findings collectively establish Cs-TPA-PiP and its Cs-TPA-PiP NPs as effective antibacterial and anti-biofilm candidates, with their activity primarily attributed to membrane disruption. The proposed role of Sortase A inhibition remains hypothetical and warrants further investigation. These findings highlight their potential as multifunctional antibacterial platforms for managing biofilm-associated and resistant bacterial infections.

## Introduction

Antimicrobial resistance (AMR) is associated with an estimated 4.95 million deaths annually and costs the global economy more than $300 billion. Biofilm production is an important survival strategy for bacteria, forming functional aggregates inside an extracellular polymeric substance (EPS) matrix made of sugars, proteins, and DNA^1^. This EPS matrix acts as a physical barrier, contributing to antibiotic resistance up to 1000-fold higher than in planktonic cells. Consequently, there is an urgent need for novel antimicrobial agents capable of disrupting biofilm integrity^2^.

The selected bacterial panel represents major clinical threats; Opportunistic Gram-negative pathogens play a significant role in oral and systemic disease and can cause acute infections. Among these pathogens, *Klebsiella* spp. are notable for their association with nosocomial infections and multidrug resistance^3^. Furthermore, *Escherichia coli* has been isolated from periodontal pockets, and its role in oral disease is attracting increasing attention. Recent studies have demonstrated that periodontal *E. coli* strains display diverse virulence genotypes, biofilm-forming capacity, multidrug resistance, and distinct phylogroup and serotype distributions. These characteristics suggest that *E. coli* may actively contribute to the pathogenesis of periodontal disease through its virulence potential and multidrug resistance profile^4^. *Pseudomonas aeruginosa* is a formidable opportunistic pathogen frequently associated with severe infections in patients with cystic fibrosis, burn wounds, and immunodeficiency. It is also a leading cause of ventilator-associated pneumonia in patients with chronic obstructive pulmonary disorder (COPD), cancer, and viral infections such as COVID-19. Furthermore, *P. aeruginosa* serves as a widely recognized model organism for studying bacterial biofilm dynamics and multidrug resistance mechanisms^5^. On the other hand, Gram-positive bacteria, including *Staphylococcus aureus*, are recognized as major human pathogens and leading cause of pyogenic infections, particularly in hospital settings, where they account for more than 80% of such diseases. *S. aureus* has been strongly associated with a range of oral conditions, including dentoalveolar infections and mucosal lesions^6^. Reported oral carriage rates vary widely—from 24% to 84% in healthy adults, and up to 48% in denture-wearing individuals. Furthermore, *S. aureus* has been implicated in several oral pathologies, including angular cheilitis, parotitis, and staphylococcal mucositis, and may even contribute to dental implant failure^7^. *Enterococcus faecalis* is also found in the oral cavity, gastrointestinal tract, and female genital tract due to its ability to thrive in nutrient-rich, low-oxygen environments. It is of particular concern in endodontics, where numerous studies have reported a higher prevalence of this organism in cases of failed root canal therapy than in primary infections. In post-endodontic disease, *E. faecalis* has been identified in up to 90.00% of cases and is more frequently associated with asymptomatic than with symptomatic primary endodontic infections^8^.

The growing need for sustainable and biocompatible materials has led to a notable increase in the field of functional biopolymers, particularly for specialized biological and medicinal applications^9,10^. Chitosan (Cs), a highly recognized natural polysaccharide, serves as a foundation for many of these materials. Chemically, chitosan is a linear polysaccharide composed of randomly distributed D-glucosamine and *N*-acetyl-D-glucosamine units linked through β−1,4-glycosidic linkages. This biopolymer, derived from chitin *via* alkaline deacetylation, possesses unique properties, including non-toxicity, biodegradability, biocompatibility, and excellent film-forming capabilities. The presence of numerous functional groups, specifically the amino (-NH_2_), primary hydroxyl (-CH_2_OH), and secondary hydroxyl (> CH-CH_2_OH) substituents, makes chitosan susceptible to chemical modification^11–13^.

Despite its inherent advantages, the practical application of unmodified chitosan is often limited by its rigidity, compact crystalline structure, and poor solubility in neutral and alkaline media^14^. Consequently, chemical derivatization is widely utilized to tailor chitosan’s physicochemical characteristics and expand its utility across diverse sectors such as wastewater purification^15^, agriculture^16^, food science^17^, and biomedicine^18^. One of the most effective strategies involves synthesizing chitosan Schiff bases (CSBs), formed by the condensation of chitosan’s free amino groups with aldehydes or ketones. The resulting CSBs incorporate the imine or azomethine linkage (> C = N-), which is known to possess good thermal stability and broad chemical reactivity^19,20^. Importantly, chemically modified derivatives like CSBs often exhibit remarkable solubility in various solvents, overcoming key limitations of the pristine polymer^21^.

The integration of specific chemical moieties into the chitosan backbone through Schiff base formation is crucial for enhancing biological efficacy. Heterocyclic compounds, which contain ring structures in which atoms other than carbon (such as N, O, or S) are part of the ring, are frequently employed as substituents in CSBs to introduce significant biological activities^22–24^. Pyrazoles, characterized as five-membered aromatic heterocycles bearing two nitrogen atoms, are electron-rich systems that serve as fertile templates for medicinal agents. Pyrazole derivatives exhibit a broad spectrum of valuable biological activities^25–27^, frequently incorporated into modern drugs due to their noted fungicidal^28,29^, antibacterial^30^, and antiviral potential^25,26^. Similarly, thiophene is an aromatic sulfur heterocycle whose derivatives are highly valued in pharmaceutical sciences and often exhibit antimicrobial activity^27^. Fused thiophene systems, such as thieno[*b*]pyridines, are recognized for their diverse pharmacological applications^28–30^, including antiviral^28^ and antidiabetic activities^31^. Complementing these structures, piperazine (C_4_H_10_N_2_), or hexahydropyrazine, is a six-membered heterocyclic compound classified as a privileged structural motif in drug discovery, containing two opposing secondary nitrogen atoms^32^. The piperazine nucleus confers critical enhancements to therapeutic agents, offering structural rigidity, a large polar surface area, and improved properties such as water solubility and oral bioavailability. Consequently, piperazine derivatives form the basis of numerous clinical drugs, including widely used antibiotics (e.g., Ciprofloxacin, Ofloxacin), anthelmintics, antifungals, and atypical antipsychotics^33,34^. The persistent challenge of microbial resistance necessitates ongoing investigation of novel compounds derived from these foundational heterocycles to generate hybrid molecules with enhanced therapeutic efficacy^24,32,35,36^.

The development of advanced drug delivery systems mandates the use of materials engineered at the nanoscale. Chitosan Schiff base nanoparticles (CSB NPs) leverage the unique properties of CSBs while capitalizing on the benefits of nanotechnology, offering attributes such as increased surface area, profound diffusion capability, and improved bioavailability of encapsulated compounds. Chitosan nanoparticles (CsNPs) are typically spherical, with diameters generally ranging from 20.00 to 200.00 nm, and are often synthesized via green ionic gelation methods using crosslinking agents such as sodium tripolyphosphate (TPP)^37–39^. The positive surface charge density of these nanoparticles facilitates interactions with biological supports, such as negatively charged cell membranes^40,41^. Recent research highlights the promise of CSB NPs and their nanocomposites (e.g., incorporating Se or Fe_2_O_3_ nanoparticles) as highly effective platforms for therapeutic interventions, including potent anti-*Helicobacter pylori* activity and cytotoxic effects against cancer cell lines^42^. Therefore, this study sought to answer the following question: Does piperazine-linked chitosan Schiff base and its nanoformulation exhibit superior antibiofilm activity compared to the bulk compound? We hypothesized that the piperazine-linked chitosan Schiff base (Cs-TPA-PiP) and its nanoformulation (Cs-TPA-PiP NPs) would exhibit potent antibacterial and antibiofilm activity. Furthermore, we hypothesized that the nanoformulation would achieve greater penetration into the biofilm than the bulk compound.

In this study, we successfully synthesized a novel ionically crosslinked chitosan–Schiff base compound, Cs-TPA-PiP, and its corresponding nanoparticles, Cs-TPA-PiP NPs, *via* the ionic gelation technique. The prepared chitosan derivatives were characterized using a range of spectroscopic techniques, including ^1^H-NMR, FTIR, and XRD spectroscopy. Furthermore, the prepared derivatives were subjected to in silico analysis to evaluate their potential antimicrobial and antibiofilm activity against pathogens implicated in oral cavity infections. The synthesized Chitosan Schiff base compound was subsequently assessed for its antimicrobial efficacy against a panel of clinically relevant bacterial species, including *Staphylococcus aureus* (ATCC 29213, 1026, and 976), *Enterococcus faecalis* (ATCC 51299 and 29212), *Klebsiella pneumoniae* ATCC 700,603, *Escherichia coli* (ATCC 35218 and 25922), and *Pseudomonas aeruginosa* ATCC 27,853.

## Materials and methods

### Materials

Shrimp shell-derived chitosan was synthesized and exhibited a viscosity-average molecular weight (Mv = 2 × 10^5 Da) and an 89.00% degree of deacetylation, as assessed by the titration method^45^. K_2_CO_3_, 2-acetylthiophene, sodium tripolyphosphate, chloroacetyl chloride, phosphorus oxychloride, and phenylhydrazine were purchased from Loba Chemie Ltd., whereas piperazine was purchased from Merck. Piochem provided acetone, glacial acetic acid, sodium hydroxide, HCl, ethanol, and DMF.

### Synthesis of 1-Phenyl-3-(thiophene-2-yl)−1 *H*-pyrazole-4-carbaldehyde

**Fig. 1:**
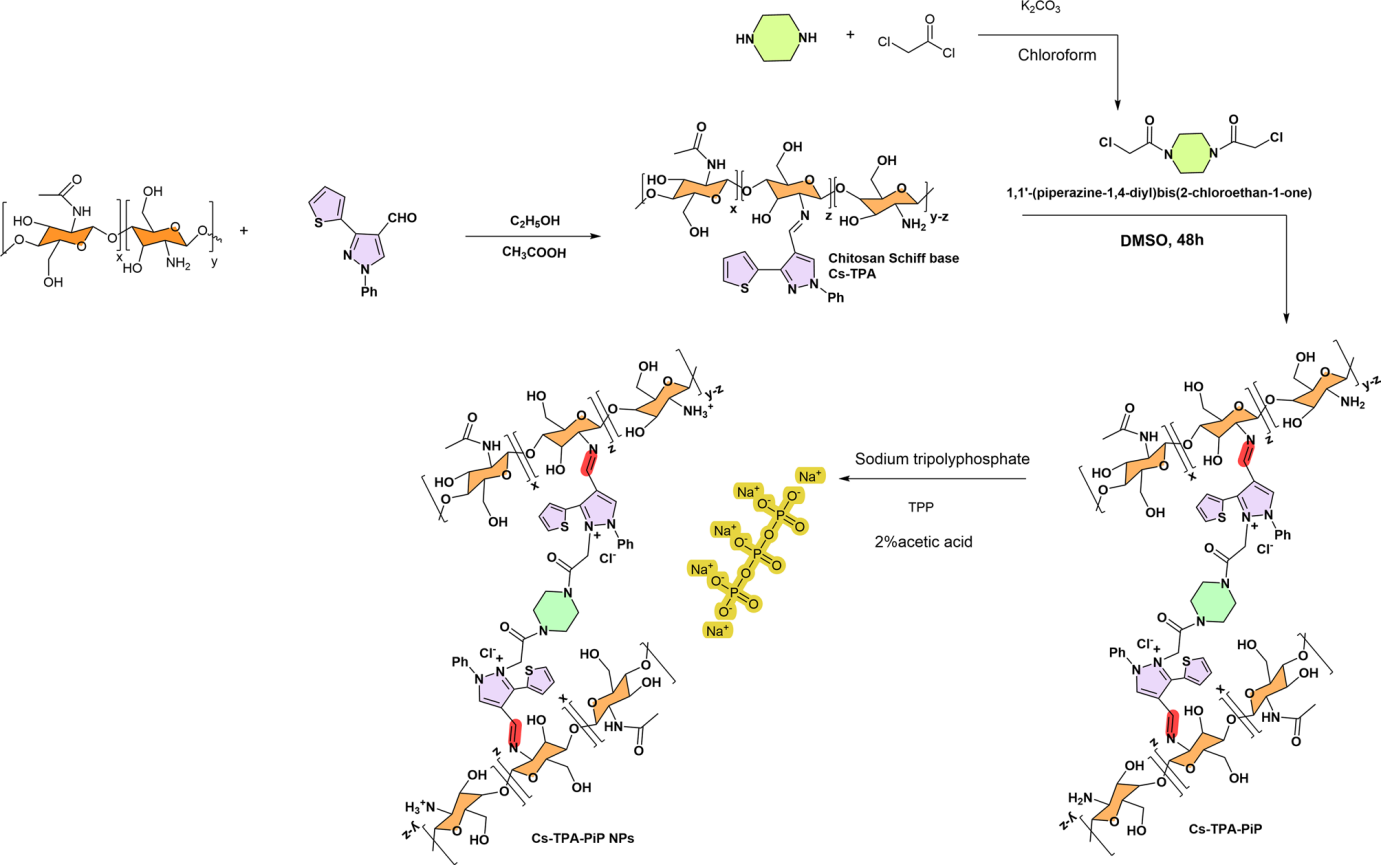
Synthesis of ionically crosslinked chitosan Schiff base and its corresponding nanoparticle derivative (Cs-TPA-PiP) and (Cs-TPA-PiP NPs).

1-Phenyl-3-(thiophene-2-yl)−1*H*-pyrazole-4-carbaldehyde was prepared using the previously demonstrated procedure^22^ in accordance with (Fig. 1). 2-acetylthiophene (0.1 mol) and phenylhydrazine (0.1 mol) were stirred in 50 mL absolute ethanol and a few drops of glacial acetic acid. The reaction mixture was refluxed for 2 h and then cooled in an ice bath. The 2-thiophene-1-phenyl-1*H*-pyrazole-5-one derivative was obtained by filtering off the product and washing it with ethanol. After that, the product was heated to 70–80 °C for five hours following the treatment with the Vilsmeier–Haack reagent (DMF-POCl_3_). After cooling at room temperature, the mixture was basified with ammonia solution. The resulting product was filtered, washed with distilled water, and crystallized from ethanol.

### Synthesis of chitosan Schiff base (Cs-TPA)

Chitosan Schiff base was produced by dissolving 2.0 g of chitosan in 150 cm³ of 2.0% aqueous acetic acid and 50 cm³ of ethanol at room temperature (25 °C). 1-Phenyl-3-(thiophene-2-yl)−1*H*-pyrazole-4-carbaldehyde was added to the solution, which was stirred constantly. The mixture was heated to 60 °C and stirred for 24 h. The final product was precipitated using ethanol and a few drops of ammonia solution to set the pH to 10. The chitosan Schiff base product was continuously washed with ethanol in a Soxhlet apparatus to ensure ultra-purification and eliminate any residual unreacted aldehydes (Fig. 1).

### Ionic crosslinking of chitosan Schiff base (Cs-TPA-PiP)

The previously outlined process was used to produce 1,1′-(piperazine-1,4-diyl)bis(2-chloroethan-1-one) (Fig. 1) with some modifications^34^. Piperazine, with a double molar ratio of potassium carbonate to chloroform, was administered dropwise for 1 h using 2-chloroacetyl chloride. The mixture was placed in an ice bath and agitated for 4 h. After 24 h at room temperature, the desired product was obtained from the organic phase after evaporation. Finally, 2.0 g of the prepared chitosan Schiff base and 2.4 g of 1,1′-(piperazine-1,4-diyl) bis(2-chloroethan-1-one) were reacted in 60 mL of DMSO, which was then agitated for 48 h at 60 °C. Resulting in crosslinked chitosan Schiff base. Excess acetone was used to precipitate Cs-TPA-PiP, which was then filtered, thoroughly washed in acetone, and boiled in ethanol to eliminate any residual unreacted 1,1′-(piperazine-1,4-diyl) bis(2-chloroethan-1-one) (Fig. 1).

### Preparation of chitosan Schiff base nanoparticles (Cs-TPA-PiP NPs)

Using sodium tripolyphosphate (TPP) as an anionic cross-linker, Cs-TPA-PiP NPs were created using the ionic gelation technique. In brief, 0.03 g of Cs-TPA-PiP was suspended in 2% acetic acid for 30 min, until complete turbidity was visible. The TPP solution was added dropwise while stirring. The reaction mixture was stirred for 2 h at room temperature. After centrifuging, the final product was repeatedly washed with distilled water, followed by acetone^43^.

### Characterization approaches

A Perkin-Elmer B25 spectrophotometer was used to acquire the FT-IR spectra of the generated Cs Schiff base derivatives. (USA, 400–4000 cm^− 1^) and a Bruker Alpha II (Germany). The ^1^H NMR spectra were recorded on a Bruker AC-400 spectrometer (Germany). Deuterated dimethyl sulfoxide (DMSO) and trifluoroacetic acid (TFA) were used as a mixture of solvents. The chemical changes were expressed in parts per million (ppm) relative to TMS as the internal reference. X-ray powder diffractograms of the generated powder samples were acquired using an X-ray powder diffractometer (Philips X’Pert MPD Pro, 50 kV/40 mA). A TA Q2000 Thermal Analyzer was used to perform thermogravimetric analysis (TGA) under nitrogen flow at a dynamic heating rate of 10 °C min^− 1^. SEM, TESCAN VEGA, and Quanta FEG 250 (FEI Company, Hillsboro, Oregon, USA) models were used to evaluate the morphological properties of Cs and its derivatives. The elemental compositions of the Cs Schiff base matrices were determined using the Quanta FEG 250 energy-dispersive X-ray (EDX). Using a JEOL (JEM 1010, Japan) transmission electron microscope (TEM), the size of the Schiff base nanoparticles was assessed, and their synthesis was validated. A Nano ZS instrument from Malvern Instrument Ltd. was used to test the zeta potential and Dynamic Light Scattering DLS (hydrodynamic size).

### Antimicrobial activity

The tested bacterial species, including *Staphylococcus aureus* (ATCC 29213, 1026, and 976), *Enterococcus faecalis* (ATCC 51299 and 29212), *Klebsiella pneumoniae* ATCC 700,603, *Escherichia coli* (ATCC 35218 and 25922), and *Pseudomonas aeruginosa* ATCC 27,853, were grown on a nutrient broth medium and incubated for 24 h at 37 °C^44^. The tested bacterial species were prepared as suspensions with a concentration equivalent to 0.5 McFarland Standard^45^. The disc-diffusion method was used to evaluate the antibacterial activity of the synthesized Cs-TPA-PiP and Cs-TPA-PiP NPs at concentrations of 1.00, 5.00, and 10.00 mg/mL, dissolved in dimethyl sulphoxide (DMSO), against the tested bacterial strains. Agar plates were inoculated with bacterial cultures and allowed to dry. Sterile 6-mm-diameter filter paper discs were impregnated with each test compound solution and placed on the inoculated agar surfaces. A negative control containing pure DMSO (10% v/v, the same concentration used for compound dissolution) showed no antibacterial activity against any of the tested strains. Standard antibiotic discs were used as positive controls: Tetracycline (30 µg/ml). The plates were incubated at 37 °C for 24 h. The diameter of the inhibition zone was measured in millimeters. All experiments were carried out in triplicate^45^.

### Measurement of minimum inhibitory concentration (MIC)

In a 96-well microtiter plate, the tested Cs-TPA-PiP and Cs-TPA-PiP NPs were dissolved in nutrient broth (NB) at double the final test concentration. The concentrations used are 0.63, 1.25, 2.50, 5.00, 10.00, and 20.00 mg/mL. Then, 5.00 µl of diluted bacterial suspension (1.5 × 10^6^ cells/ml) was thoroughly mixed with 50.00 µl of each concentration in all wells. After an overnight incubation (18–24 h) at 37 °C, 5 µl of resazurin (6.75 mg/ml) was added to all wells, and the wells were incubated at 37 °C for 2–4 h. On completion of the incubation, the MIC value is the lowest concentration of the tested compound at which no color change from blue to pink occurred^46^.

### Antibiofilm assay

#### Qualitative detection of biofilm formation

The qualitative assessment of biofilm production by the tested bacterial strains was performed using the Congo Red Agar (CRA) method. In the CRA test, bacterial cultures were streaked onto agar plates containing Brain Heart Infusion Broth (BHIB) (37.00 g/L), sucrose (50.00 g/L), agar (10.00 g/L), and Congo red dye (8.00 g/L), then incubated at 37 °C for 48 h. After incubation, biofilm-producing bacteria appeared as black colonies, whereas non-biofilm producers formed pink colonies^47^.

#### Quantitative assessment of biofilm biomass

The ability of the bacterial isolates to form biofilms was assessed using the microtiter plate assay. Fresh cultures grown on nutrient agar were inoculated into Luria-Bertani (LB) broth and incubated under static conditions at 37 °C for 18 h. The overnight cultures were diluted 1:100, and 200 µL of the diluted suspension was added to individual wells of a sterile 96-well microtiter plate as a control. For the treatment groups, the diluted bacterial suspension was incubated with the tested Cs-TPA-PiP and Cs-TPA-PiP NPs of ¼ MIC, 1/5 MIC, and MIC for 72 h at 37 °C to allow for biofilm formation. A negative control (broth without bacteria) and a positive control (bacterial culture with tetracycline at 30 mg/mL) were included. After incubation, the wells were carefully emptied by gently tapping the plates, then washed with phosphate-buffered saline (PBS, pH 7.3) to remove nonadherent (planktonic) cells. The wells were stained with 0.1% (w/v) crystal violet solution. Excess dye was rinsed off using 95% ethanol, and the plates were left to dry. Biofilm formation was quantified by measuring optical density at 600 nm (OD_600_). Each experiment was repeated three times, and the results are presented as mean ± standard error (SD)^48^.

% of inhibition = [(control OD_600_ nm−treated OD_600_ nm)/control OD_600_ nm] × 100^49^.

### Transmission electron microscopy (TEM)

Transmission electron microscopy was used to examine the ultrastructural changes in *Staphylococcus aureus* ATCC1026 cells after treatment with the Cs-TPA-PiP and Cs-TPA-PiP NPs. TEM analysis was performed using a JEM-2100 High-Resolution Transmission Electron Microscope (JEOL Ltd., Japan) operated at an accelerating voltage of 200 kV, which enables high-resolution imaging with a point resolution of approximately 0.20 nm. Bacterial cells were collected after treatment with Cs-TPA-PiP and Cs-TPA-PiP NPs at MIC and compared with untreated control cells. Morphology was assessed by TEM using a negative-staining technique. Briefly, a 5.00–10.00 µL droplet of the cell suspension was applied to a carbon-coated copper grid for 1–2 min. After removing excess liquid with filter paper, the samples were stained with 1% (w/v) phosphotungstic acid (PTA, pH 7.0) for 30–60 s, then the grids were air-dried at room temperature^50^.

### Molecular docking

Protein Preparation: The crystal structure of Sortase A (srtA) from *Staphylococcus aureus* was obtained from the Protein Data Bank (PDB) using its UniProt ID (Q2FV99). The protein structure was retrieved from the UniProt database and prepared using the ‘prepare protein’ protocol of BIOVIA Discovery Studio Visualizer 2021. Water molecules were removed, hydrogen atoms were added, and Gasteiger charges were assigned using AutoDock tools^51^. The binding sites of these proteins were predicted based on literature information and validated using CB-DOCK2. The active sites identified were used for protein-ligand interaction studies^52^. The grid boxes for docking were centered at coordinates (X=−22.667, Y=−0.056, Z = 11.778) Å with dimensions (X = 30, Y = 30, Z = 30) Å. The grid spacing was set to 0.375 Å. The exhaustiveness parameter was set to 8, and the default scoring function was used for the docking calculations^53^.

Ligand Preparation: The molecular structure of the tested compound (Cs-TPA-PiP) was drawn using the free online version of Marvin JS and then exported as an SDF file, while Fraxetin, as a positive control, was retrieved from the PubChem database in SDF format. Fraxetin, a known natural Sortase A inhibitor with experimentally validated activity in vitro and in vivo, was used as a reference compound for molecular docking^54^. Avogadro software (1.2.0) was used for energy minimization using the MMFF94 force field to avoid steric overlap and to relax the conformation^55^. These structures were then prepared using the ‘Prepare Ligand’ protocol of BIOVIA Discovery Studio Visualizer 2021.

Molecular Docking: Molecular docking is used to predict the binding orientation of small-molecule candidates to their protein targets and to estimate the binding affinity and strength of association between the target and ligand. The docking was performed between the active binding region of the structure of the microbial protein and the tested compound using the AutoDock Vina 1.5.7 tool to predict the binding modes and affinities of the tested compound with proteins of microbial species^56^.

Visualization and Analysis: The protein-ligand interactions were visualized and analyzed using BIOVIA Discovery Studio Visualizer 2021^57^. The binding affinities (ΔG values) and intermolecular interactions, including hydrogen bonds and hydrophobic interactions, were analyzed and reported. The binding affinities (ΔG values) of the ligand, in kcal/mol, were ranked negatively.

### Statistical analysis

All antimicrobial activity assays were conducted in triplicate, and results are presented as mean ± standard deviation. Statistical significance was determined using one-way ANOVA followed by Duncan’s multiple range test in SPSS (version 21), with *P* < 0.05 considered significant^58^.

## Results and discussion

### FTIR

**Fig. 2:**
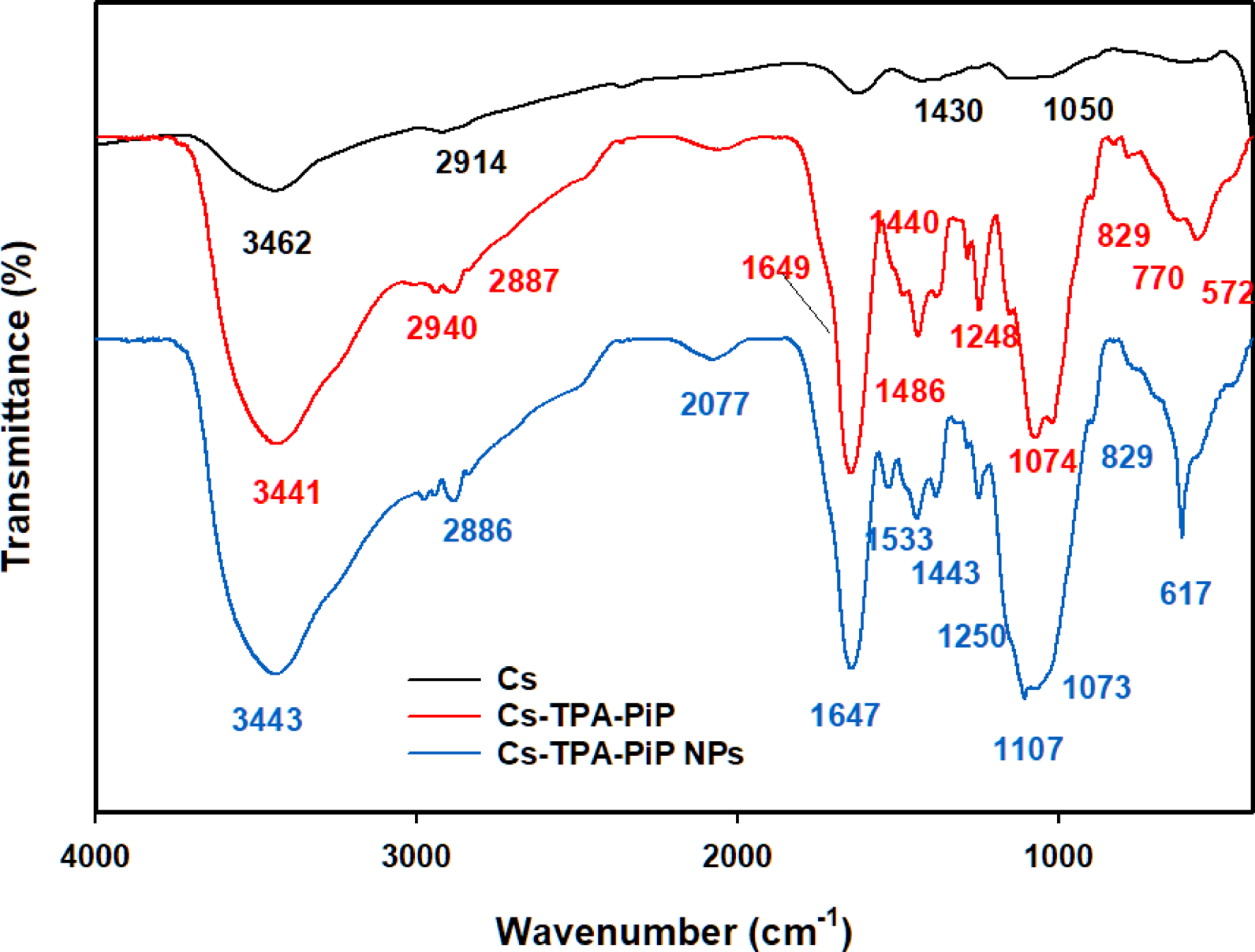
FTIR spectra of Cs, Cs-TPA-PiP, and Cs-TPA-PiP NPs.

The spectrum of pristine chitosan (Cs), as shown in Fig. 2, provides a baseline for the characteristic polymer functional groups, exhibiting a broad absorption band at 3462 cm^− 1^, characteristic of the overlap between the stretching vibrations of hydroxyl (O − H) and amino (N − H) groups^59^. The band observed at 2914 cm^− 1^ corresponds to C − H stretching vibrations. Additionally, the peak at 1050 cm^− 1^ is attributed to C − O stretching modes within the glucopyranoside ring, representing the glycosidic linkage characteristic of chitosan^60,61^.

The synthesis of the Cs-TPA-PiP derivative confirms the successful formation of the Schiff base linkage and incorporation of the heterocyclic and piperazine moieties. The spectrum shows a slight shift in the broad hydroxyl/amino region to 3441 cm − 1, consistent with chemical modification that alters the hydrogen-bonding network. Crucially, the peak observed at 1649 cm^− 1^ is attributed to the stretching vibration of the new imine functional group (> C = N−), confirming the condensation reaction between the amino groups of chitosan and the aldehyde functional groups present in the 1-Phenyl-3-(thiophene-2-yl)−1*H*-pyrazole-4-carbaldehyde (TPA)^22^. The presence of the phenyl and pyrazole/thiophene heterocyclic structures is confirmed by characteristic aromatic stretching modes. Bands observed at 1486 cm^− 1^ and 1440 cm^− 1^ fall within the 1430–1625 cm^− 1^ range associated with C = C ring stretching vibrations of aromatic systems^62,63^. Direct evidence for the thiophene subunit is furnished by the presence of the absorption peak at 829 cm^− 1^, which is characteristically ascribed to the C-S-C stretching vibration inherent to the thiophene moiety^22,64^. Furthermore, proof of the piperazine inclusion is supported by multiple signals. Aliphatic C-H stretching vibrations from both the chitosan backbone and the methylene (CH_2_) units of the piperazine ring are clearly observed at 2940 cm^− 1^ and 2887 cm^− 1^^65^. Crucially, the peaks at 1285 cm^− 1^ and 1248 cm^− 1^ corroborate the presence of the piperazine component, aligning well with the typical range of 1000–1342 cm^− 1^ for C-N stretching vibrations specific to the piperazine ring structure^34,66,67^. The spectrum of the Cs-TPA-PiP NPs confirms the formation of nanoparticles *via* ionic gelation using sodium tripolyphosphate (TPP)^68^. The core Schiff base structure is maintained, as evidenced by the persistent imine peak at 1647 cm^− 1^. The most significant indicator of ionic crosslinking is the appearance of a strong absorption band at 1533 cm^− 1^^69–71^. This prominent peak is assigned to the N − H bending vibration of the protonated primary amino groups (NH_3_^+^), confirming the electrostatic interaction between the cationic chitosan derivative and the anionic polyphosphoric moiety of TPP. Furthermore, the introduction of the TPP crosslinker is explicitly supported by the presence of new peaks related to phosphate functional groups, observed prominently at 1250 cm^− 1^ and 1107 cm^− 1^, corresponding to the asymmetric and symmetric stretching vibrations of phosphate (P = O and P − O) in the nanoparticle matrix, verifying the structural changes essential for nanoparticle formation^72,73^.

**Fig. 3:**
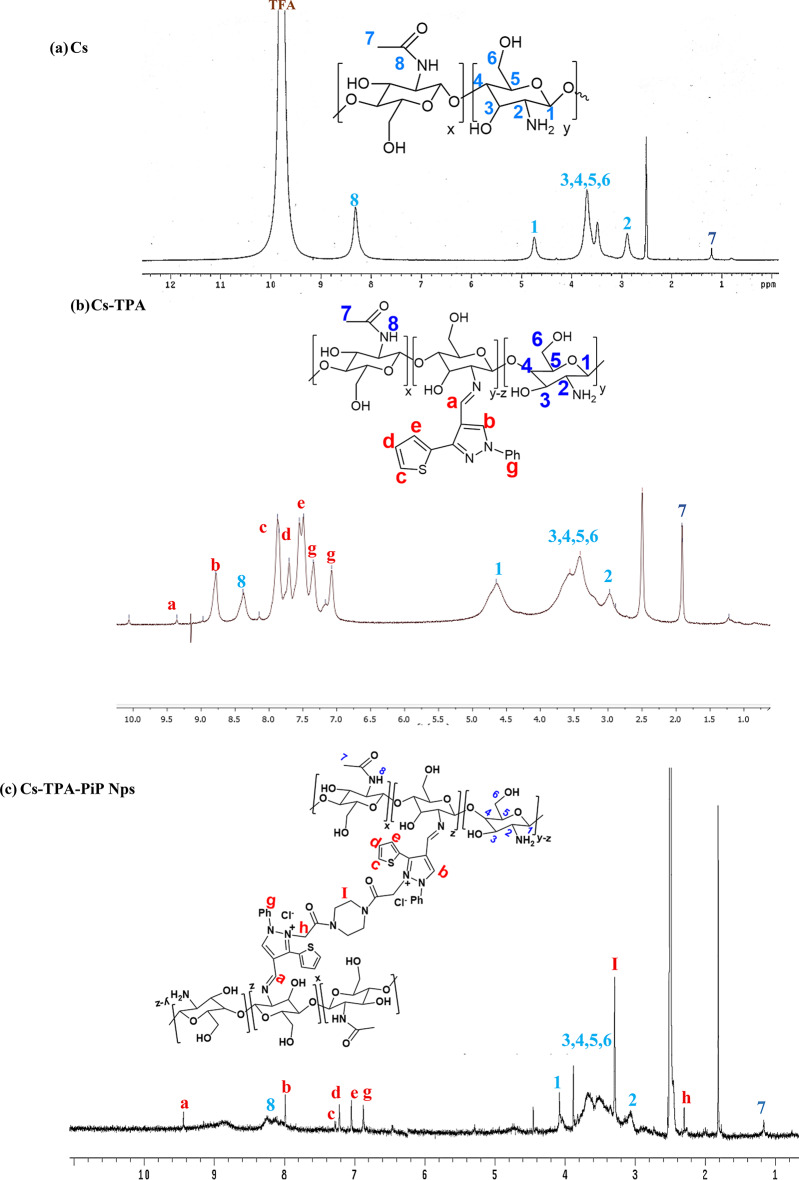
^1^HNMR spectra of (**a**) Cs (**b**)Cs-TPA, and (**c**) Cs-TPA-PiP NPs.

Pristine chitosan (Cs) showed signals consistent with the polymer backbone in its ^1^H NMR spectrum (Fig. 3). These distinctive signals include the H − 2 proton of the pyranose ring, which appears at 2.9 ppm, and the *N*-acetyl CH_3_ protons, which are usually detected around 1.8 ppm. The additional skeletal protons (H − 3, H − 4, H − 5, and H − 6) are usually detected in a multiplet between 3.4 and 4.7 ppm^74–76^. Exchangeable protons, such as those from hydroxyl (O − H) or residual amine (N − H) groups remaining in the polysaccharide structure, have frequently been attributed to the large peak seen at 8.29 ppm in the chitosan^77,78^. The incorporation of the therapeutic heterocyclic components was confirmed by signals in the downfield region characteristic of aromatic and imine protons. The signals observed at 6.9 ppm, 7.0 ppm, 7.2 ppm, and 7.9 ppm, along with a broad peak around 8.2 ppm, confirm the presence of the aromatic rings associated with the TPA moiety (phenyl and thiophene units)^79,80^. The successful formation of the Schiff base linkage (imine proton, -N = CH-) resulting from the condensation reaction between the amino groups of chitosan and the TPA aldehyde group is supported by the distinct signal observed at 9.4 ppm, which falls within the typical downfield chemical shift range reported for imine protons in similar chitosan derivatives^22^. Furthermore, the successful integration of the piperazine (PiP) core was demonstrated by new aliphatic signals. The peaks observed at 3.1, 3.3, and 3.35 ppm, and the broader multiplet stretching from 3.5 − 3.6 ppm are definitively assigned to the methylene protons (N−CH_2_) located within the piperazine ring system. Such signals confirm the grafting of the 1,1′-(piperazine-1,4-diyl)bis(2-chloroethan-1-one) linker onto the chitosan structure^34,65,81^. The degree of substitution DS of Cs-TPA-PiP NPs was determined from the ^1^H NMR spectrum using Eq. (1) and was found to be 0.13.

1 $$\:DS={A}_{H-a}/{(A}_{H-a}+{A}_{H-2})$$

Where A_H−a_ is corresponding to the region of the proton associated with the imine moiety in the.

^1^H NMR spectrum and A_H−2_ denotes the region of the proton associated with H-2 of the pyranose ring in chitosan^82,83^.

### XRD

**Fig. 4:**
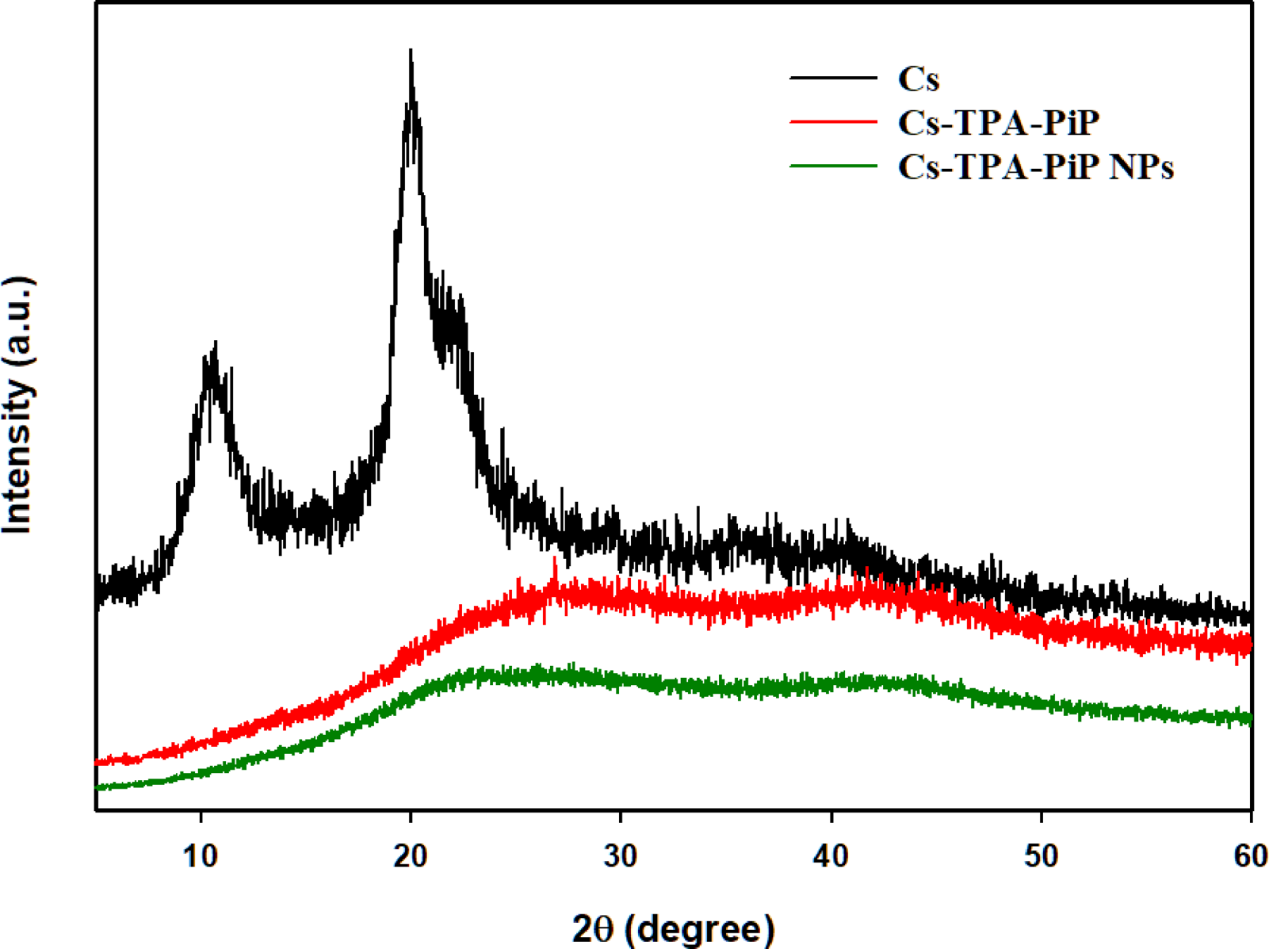
XRD patterns of Cs, Cs-TPA-PiP and Cs-TPA-PiP NPs.

The spectrum of pristine chitosan (Cs) exhibited a semi-crystalline character, displaying two prominent diffraction peaks around 10° and 20° (2*θ*), which are typically indexed to the (020) and (110) planes of the polymer’s crystalline lattice^78,84^ as shown in Fig. 4. Upon conversion to the chitosan Schiff base derivative, Cs-TPA-PiP, the original sharp crystalline peaks of pristine chitosan almost disappeared and were replaced by notably broad, amorphous signals appearing around 27° and 41° (2*θ*). This shift confirms the successful grafting of the bulky heterocyclic moieties (pyrazole/thiophene) and the piperazine functional group onto the chitosan backbone, a modification process that severely disrupts the inter- and intramolecular hydrogen bonding network of the native polysaccharide structure^85^. Consequently, the Cs-TPA-PiP derivative displays a highly amorphous structure due to the increased steric hindrance introduced by the voluminous substituents. The resulting Cs-TPA-PiP NPs derived *via* ionic gelation with sodium tripolyphosphate (TPP) displayed an XRD pattern similar to that of the parent Cs-TPA-PiP derivative but with decreased intensity. The maintenance of this broad, amorphous profile further substantiates that the ionic cross-linking step reinforced the non-crystalline nature of the structure. The overall reduction in peak intensity in the Cs-TPA-PiP NPs spectrum further indicates a highly disordered structure, consistent with the successful formation of the corresponding nanoparticle derivative.

### TGA

**Fig. 5:**
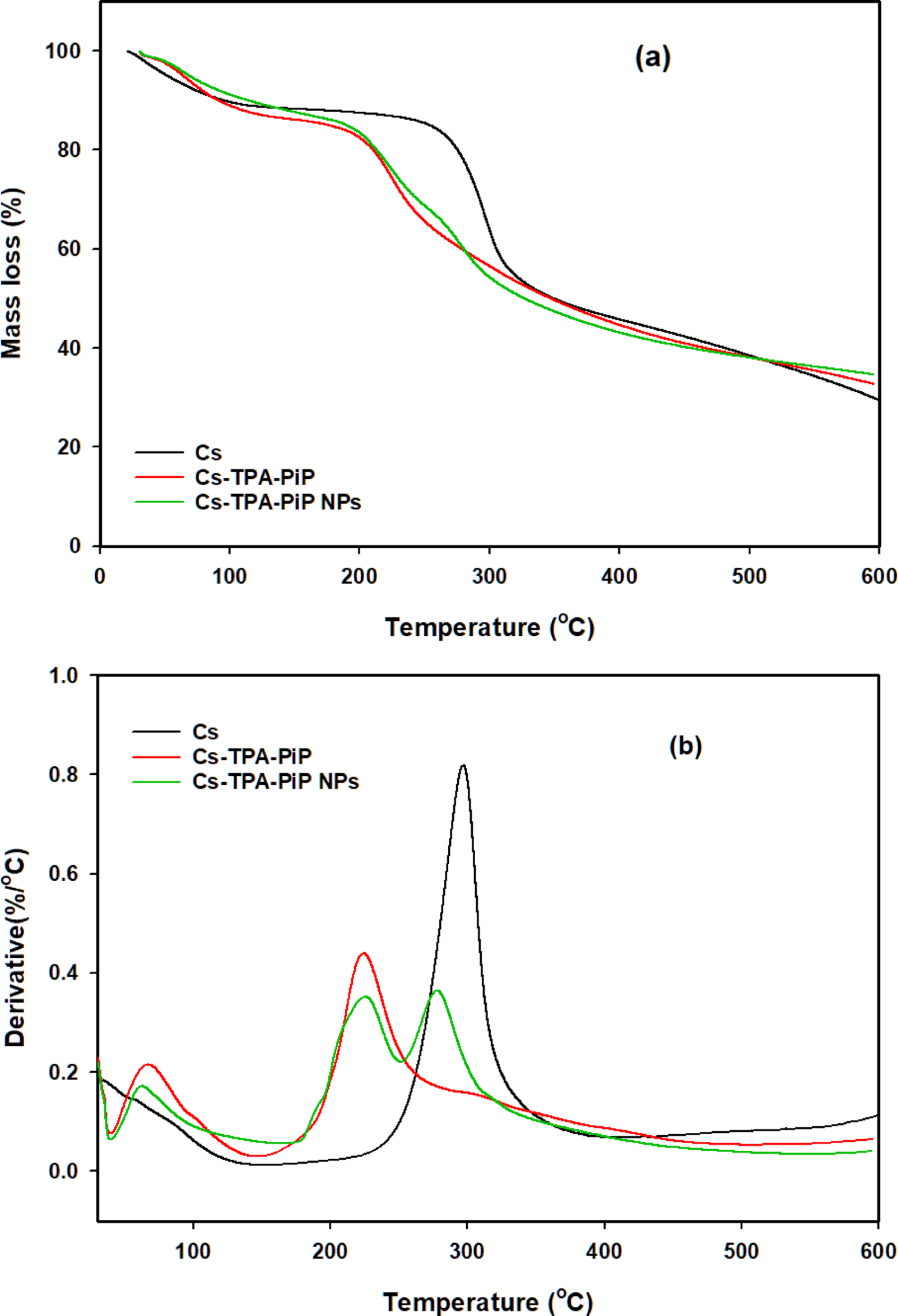
TGA (**a**) and DTGA (**b**) of chitosan (Cs), Cs-TPA-PiP, and Cs-TPA-PiP NPs.

The thermogram of pristine chitosan (Cs) provides the reference profile, showing a significant single degradation event in the main decomposition temperature range of 226 to 370°C with a mass loss of 38.8% as shown in Fig. 5. This stage is attributed to the thermal degradation and chain scission of the D-glucosamine units of the chitosan backbone, consistent with known degradation behavior for the polysaccharide^86,87^. Cs-TPA-PiP exhibited a distinct profile with two major mass-loss stages. The first stage occurred between 39 and 135°C with a mass loss of 11.4%, representing the loss of physically adsorbed moisture. The second principal decomposition stage occurred from 164 to 373 °C with a mass loss of 38.5%. This stage typically corresponds to the cleavage of the imine linkage, the degradation of the heterocyclic and piperazine groups bound to the chitosan chain, and subsequent depolymerization of the chitosan backbone itself^88,89^.

Cs-TPA-PiP NPs presented a three-stage decomposition profile. The first stage occurred between 39 to 111°C with a mass loss of 7.8%, attributed to the evaporation of adsorbed moisture. Notably, the initial mass-loss onset temperature (around 177°C) is lower than that of Cs (226°C) and comparable to that of the Cs-TPA-PiP derivative (164°C), suggesting that derivatization and nanoparticle formation increase the sample’s degradation at low temperatures relative to pristine chitosan. The second stage spanned 177 to 250°C with a mass loss of 17.4%, followed by a third stage from 251 to 348°C with a mass loss of 21%^41,42^. The shift in the main degradation profile to lower temperature ranges (starting at 177°C) compared to pristine chitosan (starting at 226°C) is a common indicator of successful functionalization and nanoparticle formation, demonstrating that the chemical modification and subsequent ionic crosslinking with TPP generally reduces the thermal stability of the polymer structure^19,90^.

**Fig. 6:**
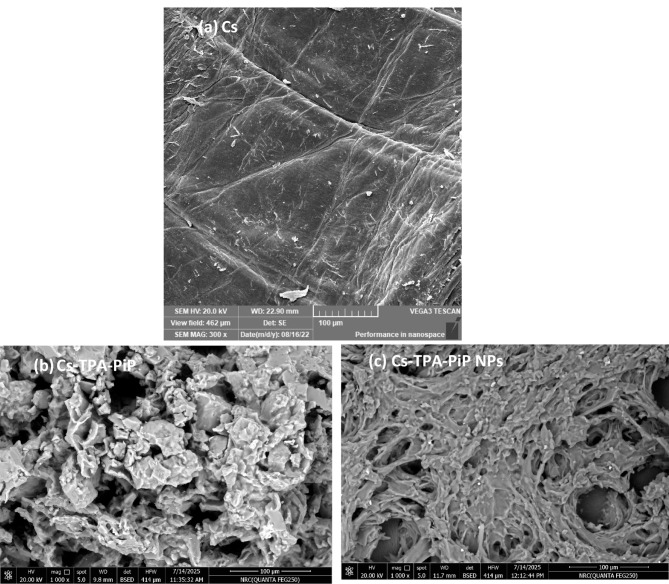
SEM images of (**a**) chitosan (Cs), (**b**) Cs-TPA-PiP, and (**c**) Cs-TPA-PiP NPs.

**Fig. 7:**
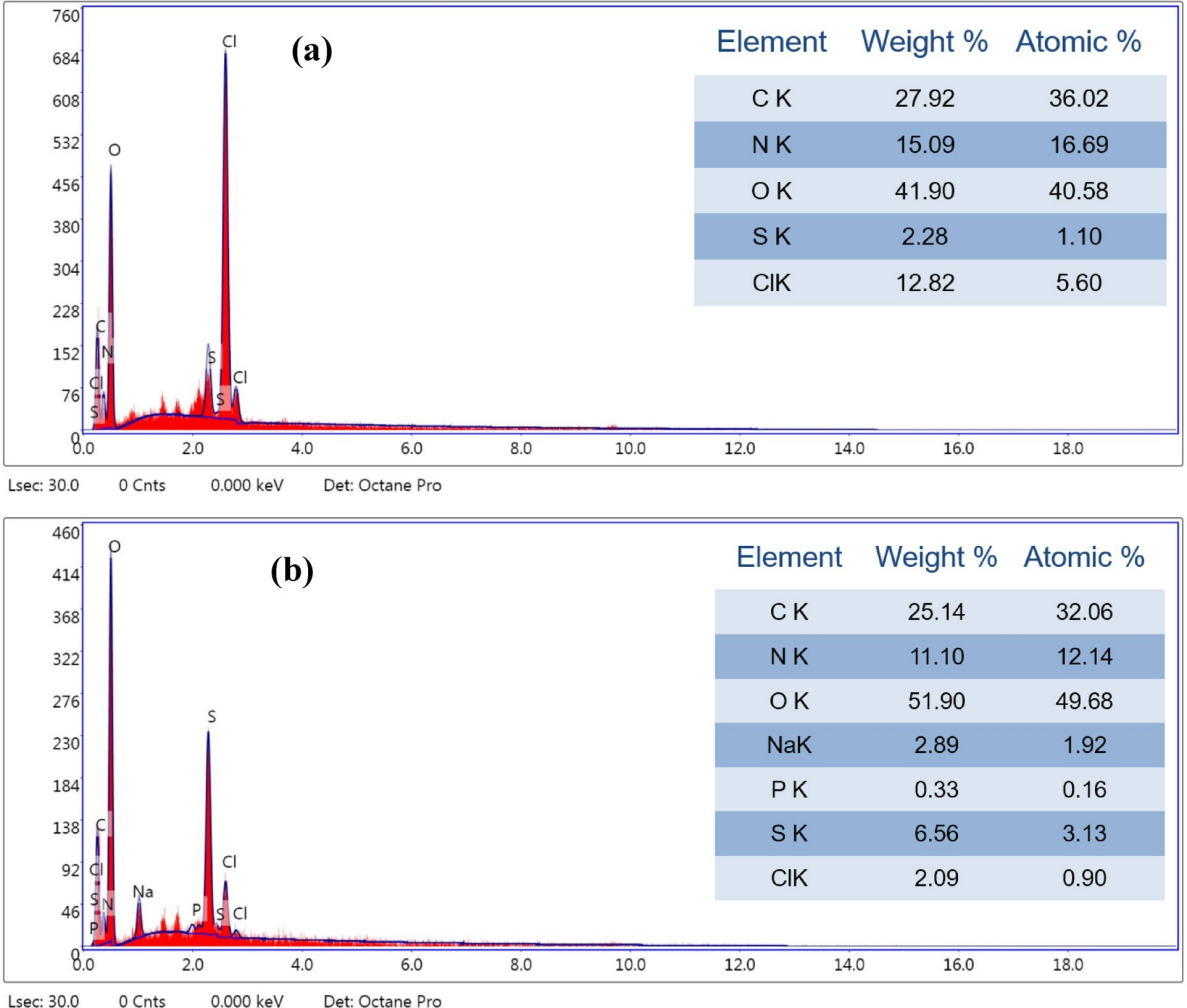
EDX spectra of (**a**) Cs-TPA-PiP and (**b**) Cs-TPA-PiP NPs.

### SEM/EDX

The SEM image of pristine chitosan (Cs) exhibited a characteristic compact, smooth surface morphology, consistent with the well-known appearance of unmodified polysaccharide structures^91^. Upon conversion to the chitosan Schiff base derivative, Cs-TPA-PiP, the SEM micrograph displayed notable irregularities and surface roughness, indicating a significant disruption of the ordered hydrogen bonding network found in native chitosan due to the incorporation of bulky substituents as shown in Fig. 6. The corresponding EDX spectrum confirmed the successful grafting of the precursors by identifying (C), (O), and (N), accompanied by characteristic signals for (S) (originating from the thiophene moiety of TPA) and (Cl) (derived from the piperazine linker, PiP). The subsequent formation of the nanoparticle derivative, Cs-TPA-PiP NPs, employed ionic gelation with sodium tripolyphosphate (TPP), yielding a surface morphology that was more uniform than that of the intermediate Cs-TPA-PiP derivative. This morphological transition is consistent with the formation of defined nanoscale structures. The EDX analysis of the Cs-TPA-PiP NPs spectrum retained the key elements (C, O, N, S, and Cl), confirming the stability of the functionalized polymeric backbone. Furthermore, the EDX spectrum detected new signals for (P) and (Na), providing definitive elemental evidence of successful ionic crosslinking and the resulting nanocomposite formation using sodium tripolyphosphate (Fig. 7).

### TEM

**Fig. 8:**
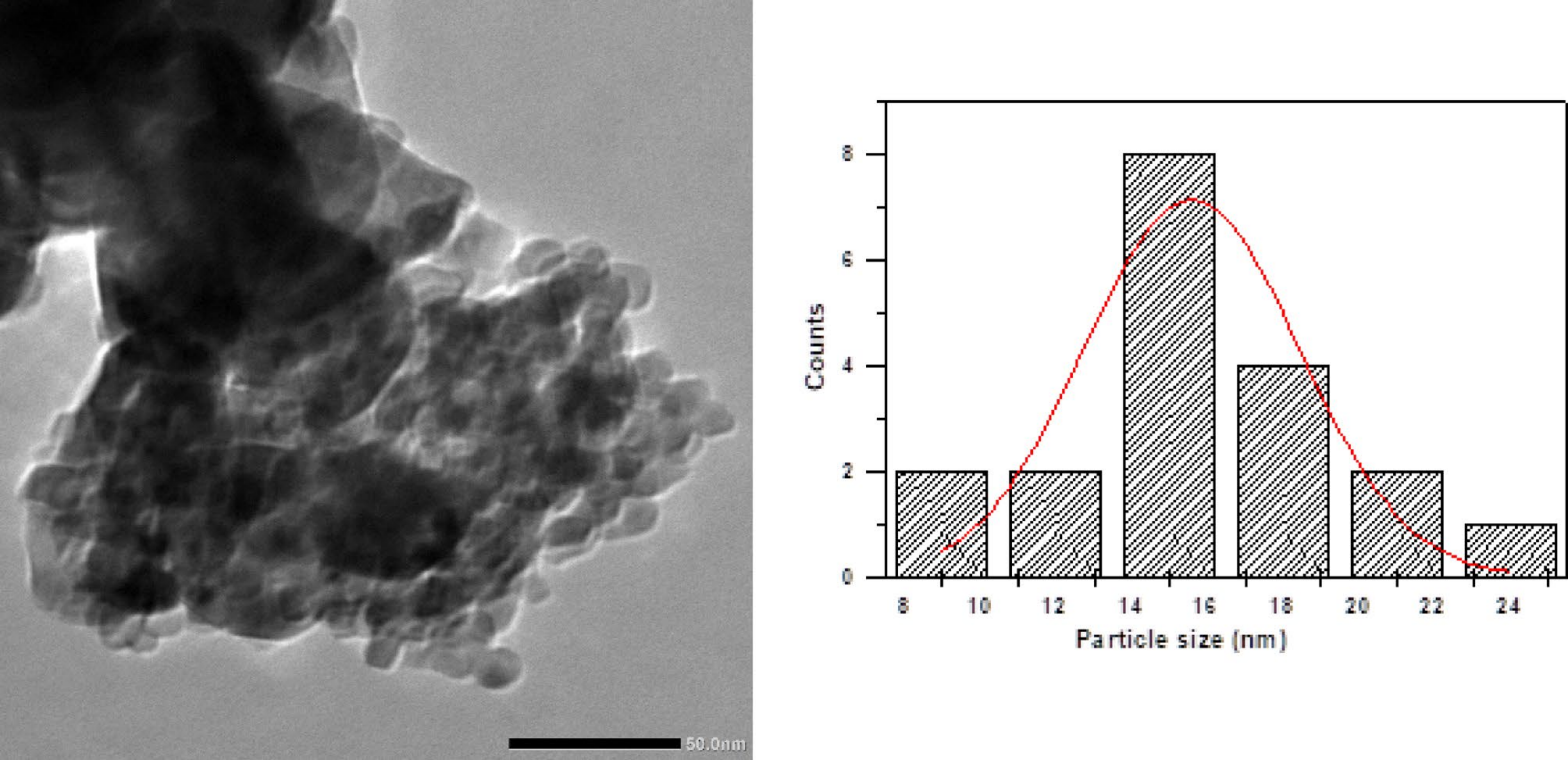
TEM image of Cs-TPA-PiP NPs.

TEM analysis (Fig. 8) revealed that the chitosan Schiff base nanoparticles (Cs-TPA-PiP Nps) exhibited a spherical morphology with an average size of 15.6 nm. This nanometric scale is consistent with typical results for nanoparticles produced *via* this technique, which often range from tens to hundreds of nanometers^92,93^. The observed small size (15.6 nm) is notably favorable, as the ionic gelation method is valued for its ability to generate systems with low particle diameters^68,94^. The resulting visualization of a layered spherical structure suggests a highly condensed, compact internal matrix, arising from intensive inter- and intramolecular cross-linkages formed during the ionic cross-linking process, as shown in Fig. 8.

### Zeta potential and dynamic light scattering (DLS) study

**Fig. 9:**
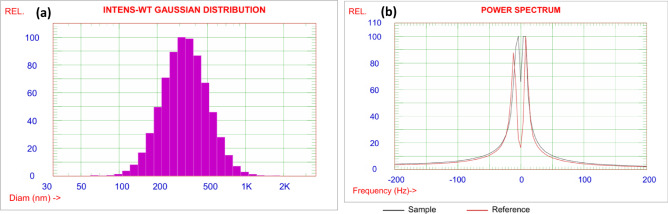
Particle size distribution (**a**) and Zeta potential (**b**) of Cs-TPA-PiP NPs.

The characterization of the Cs-TPA-PiP NPs revealed a zeta potential of −4.3 mV, a DLS hydrodynamic size of 360.1 nm in distilled water, and a polydispersity index (PDI) of 0.17 as shown in (Fig. 9). While TEM analysis demonstrated that the primary nanoparticles possess a spherical morphology with an average diameter of approximately 15.6 nm, the significantly higher hydrodynamic size (360.1 nm) and the low zeta potential provide evidence of nanoparticle aggregation in the suspension^95,96^. The measured zeta potential of −4.3 mV falls within the instability zone, where the net electrical charge on the nanoparticle surface is near the isoelectric point^97^. At this potential, electrostatic repulsive forces are insufficient to overcome attractive Van der Waals forces, leading to physical instability and a high propensity for rapid aggregation or flocculation. This confirms successful Schiff base functionalization, as the conversion of primary amines into imine linkages reduces the available sites for protonation and effectively masks the residual surface charge of the polymer backbone. The difference between the TEM result (15.6 nm) and the DLS measurement (360.1 nm) is attributed to the hydrodynamic diameter measurement, which includes the nanoparticle and its associated solvation/hydration layer in an aqueous environment^98–100^. In addition, DLS identifies aggregates or clusters of smaller particles as a single large particle^96^. Moreover, the PDI of 0.17 indicates a relatively narrow and homogeneous size distribution of these formed clusters within the suspension. These findings fulfill the requirement for verified surface properties, as the combination of near-zero zeta potential and large hydrodynamic size provides a direct empirical link between the chemical modification of the chitosan and the physical aggregation behavior of the resulting nanoformulation^100^.

### Antimicrobial activity

**Fig. 10:**
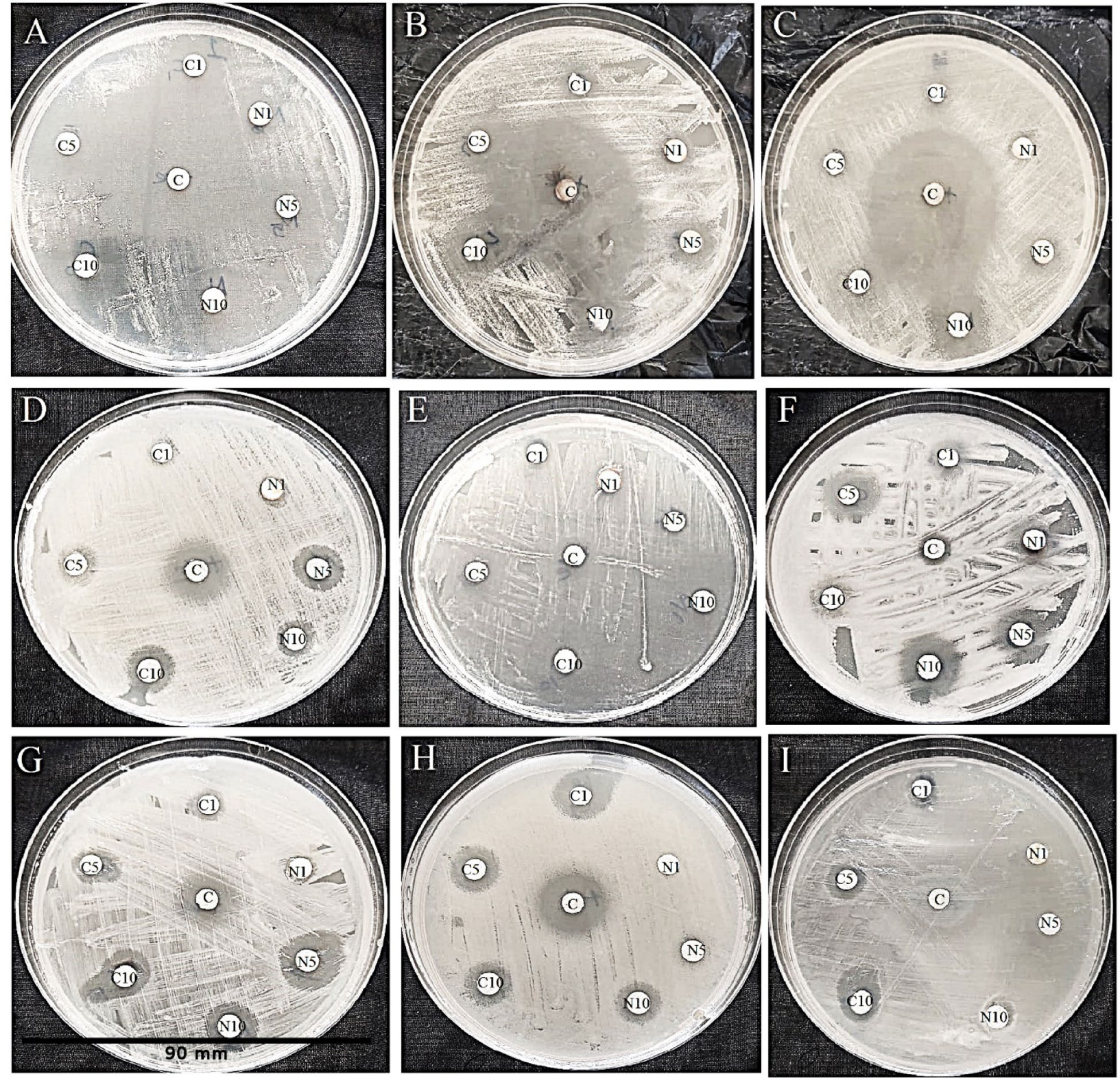
Antimicrobial activity (inhibition zone diameters in mm) of the Cs-TPA-PiP and Cs-TPA-PiP NPs against (A-C) *S. aureus* ATCC 29,213, 1026, 976, (D, E) *E. faecalis* ATCC 29,212, 51,299, (F) *K. pneumoniae* ATCC 700,603, as (G, H) *E. coli* ATCC 35,218, 25,922, and (I) *P. aeruginosa* ATCC 27,853. C: positive control (tetracycline), C1, C5, C10: Chitosan Schiff base at concentrations of 1.00, 5.00, and 10.00 mg/mL, respectively. N1, N5, N10: Chitosan Schiff base nanoparticles at concentrations of 1.00, 5.00, and 10.00 mg/mL, respectively. Scale bar = 90.00 mm.

The tested Cs-TPA-PiP and Cs-TPA-PiP NPs at the concentrations of 1.00, 5.00 and 10.00 mg/ml exhibited antibacterial activities against *Staphylococcus aureus* (ATCC 29213, 1026, and 976), *Enterococcus faecalis* (ATCC 51299 and 29212), *Klebsiella pneumoniae* ATCC 700,603, *Escherichia coli* (ATCC 35218 and 25922), and *Pseudomonas aeruginosa* ATCC 27,853, as shown in Table 1; Fig. 10. In addition, tetracycline (30.00 mg/ml) was used as a standard for antibacterial activity, their antibacterial activities increased with increasing concentration. The antimicrobial activity of Cs-TPA-PiP may be attributed to the presence of piperazine, as reported by Jalageri et al.^33^ who stated that piperazine causes cell lysis, disrupts cellular functions, and alters membrane integrity. Piperazine has been used to create highly effective sixth-generation antibiotics and is considered a valuable starting point for developing new drugs. Piperazine provides a large polar surface area, structural rigidity, and hydrogen-bond acceptors and donors, which often lead to enhanced target affinity, specificity, water solubility, oral bioavailability, and ADME (i.e., absorption, distribution, metabolism, and excretion) properties^101^.

In the present study, the Cs-TPA-PiP demonstrated **s**ignificant antibacterial activity at 1.00 mg/mL against *S. aureus* ATCC 29,213, producing an inhibition zone of 14.67 ± 0.33 mm, the highest observed among all tested strains. This indicates that chitosan derivatives, particularly Schiff bases, exhibit enhanced antibacterial activity, likely due to structural modifications that enhance interactions with bacterial cell membranes^102^. The modified Schiff base structure may interact more effectively with the cell envelopes of certain strains but less so with others, underlining the importance of molecular interactions in determining antimicrobial potency^103^. The observed spectrum of activity, including the notable variation in susceptibility among the tested *S. aureus* strains, aligns with the well-recognized phenomenon in antibacterial studies of chitosan derivatives. This variation is primarily attributed to inherent differences in bacterial cell wall structure and permeability, which govern compound interaction and diffusion. Therefore, structural modifications in the compound that promote hydrophobic interactions and improve charge balancing can differentially influence its efficacy against various strains^103^.

At a concentration of 10.00 mg/mL, the tested Cs-TPA-PiP exhibited significant antibacterial activity, with notably greater potency against Gram-positive than against Gram-negative strains. This was demonstrated by inhibition of Enterococcus faecalis ATCC 51,299 and Staphylococcus aureus ATCC 29,213, yielding inhibition zone diameters of 23.33 ± 0.88 mm and 21.33 ± 1.2 mm, respectively. The observed variation in antimicrobial activity among the tested bacteria is due to Gram-positive bacteria typically exhibiting higher susceptibility to antimicrobial agents than Gram-negative strains. This differential sensitivity is fundamentally rooted in their distinct cell wall architecture. While Gram-negative bacteria possess a complex outer membrane that acts as a selective permeability barrier, the thick but porous peptidoglycan layer of Gram-positive bacteria allows for more efficient penetration and accumulation of the tested compound^104^. Introduction of aromatic aldehyde-derived Schiff substituents increased antibacterial potency, consistent with enhanced hydrophobic interactions and membrane perturbation^105^. In addition, thiophene-pyrazole derivatives act as excellent whole-cell efflux pump inhibitors in mycobacterial surrogates, thereby increasing intracellular retention of antibacterial species^106^.

At a concentration of 1.00 mg/mL, the Cs-TPA-PiP NPs exhibited clearly differential antibacterial activity among the tested strains. *K. pneumoniae* ATCC 700,603 showed the highest susceptibility, with an inhibition zone of 13.00 ± 1.15 mm. Interestingly, further increasing the concentration did not yield a proportional expansion of the inhibition zones for this specific strain, reaching only 14.00 ± 0.58 mm at 5 mg/mL and 15.33 ± 1.2 mm at 10 mg/mL. This effect suggests that the maximum inhibitory potential against *K. pneumoniae* is achieved at relatively low concentrations, possibly due to the early saturation of bacterial surface binding sites by the nano-compound. The antibacterial effects of synthesized Cs-TPA-PiP NPs against *K. pneumoniae* and *P. aeruginosa* may be due to the physicochemical properties of the nanoparticles, such as surface charge and particle size, which can facilitate interaction with bacterial cell envelopes at elevated concentrations. These interactions likely contribute to robust inhibition of susceptible strains such as *E. faecalis*^107^. In contrast, other strains, including *S. aureus* ATCC 976, *E. faecalis* ATCC 51,299, *E. coli* ATCC 35,218, and *E. coli* ATCC 25,922, showed no inhibition at 1.00 mg/mL. A similar lack of activity was observed for *P. aeruginosa* ATCC 27,853 at both 1.00 and 5.00 mg/mL. However, a significant increase in potency was observed for *P. aeruginosa* at 10.00 mg/mL, yielding an inhibition zone of 7.67 ± 0.67 mm. The antibacterial efficacy of Cs-TPA-PiP NPs generally increases with higher concentrations, and they can inhibit both Gram-negative and Gram-positive bacteria, though with variable sensitivity across species^108^. However, the observed lack of inhibition against *P. aeruginosa* at 5.00 mg/mL in the present study suggests that this strain may exhibit features that reduce susceptibility to nanoparticle uptake or interaction at this concentration, such as efflux mechanisms or a more robust outer membrane. Essa et al. (2022) showed that *P. aeruginosa* can exhibit lower sensitivity to chitosan nanoparticle systems than other Gram-negative pathogens under similar testing conditions^107^. The highest susceptibility of Cs-TPA-PiP NPs at 10 mg/mL was observed in *Enterococcus faecalis* ATCC 51,299, with an inhibition zone of 28.33 ± 0.88 mm, followed by *Staphylococcus aureus* ATCC 29,213, which exhibited a zone of 18.33 ± 0.89 mm. Chitosan nanoparticle systems have been shown to possess inherent antimicrobial activity, which may interact more effectively with certain bacterial envelopes than with others. The presence of charged amino groups and increased surface area in nano-formulations enhances electrostatic interactions with bacterial cell walls, facilitating cell membrane disruption and growth inhibition^107^.

**Table 1:** Antimicrobial activity represented by the inhibition zone (mm) of the tested Chitosan Schiff base (Cs-TPA-PiP) and Chitosan Schiff base nanoparticles (Cs-TPA-PiP NPs) at different concentrations (1.00, 5.00, and 10.00 mg/mL).

Bacterial species	Chitosan Schiff base Cs-TPA-PiP			Chitosan Schiff base nanoparticles Cs-TPA-PiP NPs			Tetracycline (30.00 µg/ml)
	1.00 mg/ml	5.00 mg/ml	10.00 mg/ml	1.00 mg/ml	5.00 mg/ml	10.00 mg/ml	
*S. aureus* ATCC 29,213	14.67 ± 0.33	19.00^c^ ± 1.53	21.33^d^ ± 1.2	11.67^c^ ± 0.33	15.00^e^ ± 0.58	18.33^d^ ± 0.88	34.33^d^ ± 1.2
*S. aureus* ATCC 1026	10.33^d^ ± 0.88	12.33^ab^ ± 1.76	16.33^bc^ ± 2.03	11.67^c^ ± 1.2	14.00^de^ ± 0.58	15.33^bcd^ ± 2.33	36.00^d^ ± 2.31
*S. aureus* ATCC 976	7.33^a^ ± 0.33	9.00^a^ ± 1.15	11.33^a^ ± 0.67	0.00^a^ ± 0	12.00^bc^ ± 0.58	15.67^cd^ ± 0.33	32.67^d^ ± 1.2
*E. faecalis* ATCC 29,212	8.33^abc^ ± 0.33	10.00^a^ ± 0.58	14.00^abc^ ± 1.15	8.33^b^ ± 0.88	11.00^c^ ± 1.15	11.67^bc^ ± 2.03	15.00^b^ ± 0.58
*E. faecalis* ATCC 51,299	9.00^abcd^ ± 0.58	14.67^b^ ± 0.88	23.33^d^ ± 0.88	0.00^a^ ± 0	12.67^cde^ ± 1.45	28.33^e^ ± 0.88	12.00^a^ ± 0
*K. pneumoniae* ATCC 700,603	9.33^bcd^ ± 0.33	12.67^ab^ ± 1.2	12.67^ab^ ± 2.03	13.00^c^ ± 1.15	14.00^d^ ± 0.58	15.33^bcd^ ± 1.2	9.33^a^ ± 0.88
*E. coli* ATCC 35,218	8.00^ab^ ± 0.58	9.67^a^ ± 0.33	13.67^abc^ ± 0.88	0.00^a^ ± 0	10.67^c^ ± 0.88	16.33^d^ ± 0.88	12.33^ab^ ± 1.2
*E. coli* ATCC 25,922	10.00^cd^ ± 0.58	12.33^ab^ ± 0.33	17.33^c^ ± 0.88	0.00^a^ ± 0	7.67^b^ ± 0.33	11.33^ab^ ± 0.88	20.00^c^ ± 1.15
*P. aeruginosa* ATCC 27,853	9.33^bcd^ ± 0.88	10.00^a^ ± 1.15	12.67^ab^ ± 0.88	0.00^a^ ± 0	0.00^a^ ± 0	7.67^a^ ± 0.67	9.67^a^ ± 0.33
LSD (0.05)	0.87	1.34	1.72	2.29	1.79	2.3	4.25

Data are expressed as mean ± standard deviation (*n* = 3). Different lowercase letters within the same column indicate statistically significant differences (*p* < 0.05). The positive control (tetracycline) was used.

### Measurement of minimum inhibitory concentration (MIC)

**Fig. 11:**
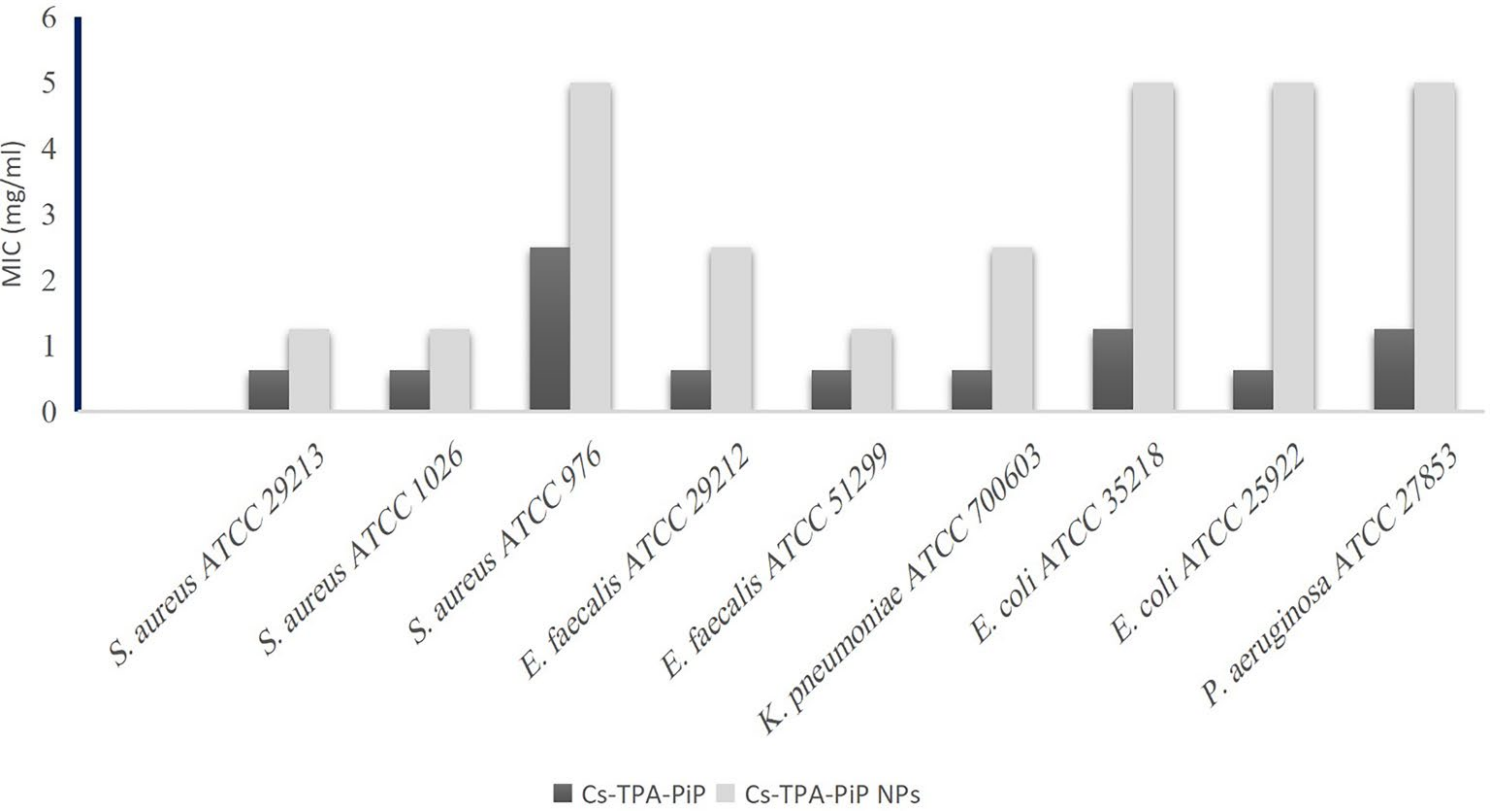
Minimum inhibitory concentration (MIC) of the Cs-TPA-PiP and Cs-TPA-PiP NPs.

The Minimum inhibitory concentration (MIC) of the Cs-TPA-PiP ranged from 0.63 to 2.5 mg/mL, while that of its Cs-TPA-PiP NPs derivative ranged from 1.00 to 5.00 mg/mL against the tested bacterial strains (Fig. 11). Interestingly, in the present study, the MIC values of the Cs-TPA-PiP NPs were found to be consistently higher than those of the conventional Cs-TPA-PiP across all tested bacterial strains. One plausible explanation is that nanoparticle formulation can alter the material’s physicochemical properties in ways that do not necessarily enhance its antibacterial effectiveness. Unlike some optimized nano-systems designed to improve bacterial cell interactions, poorly stabilized or aggregated nanoparticles can reduce effective surface area and lead to non-uniform interactions with microbial membranes, thereby requiring higher concentrations to achieve inhibition. Aggregation in suspension is a common issue that reduces the number of available active particles, thereby increasing the apparent MIC^109^. Furthermore, the degree of nanoparticle aggregation and alterations in surface charge distribution critically influence interactions with the bacterial envelope. While well-dispersed nanoparticles with a high positive zeta potential can exhibit enhanced activity through strong electrostatic attraction, a poorly optimized nanoform may lose these advantages, necessitating higher MICs to achieve the same inhibitory effect^110^. Although nanosizing is generally pursued to improve drug delivery and diffusion, its success is contingent on the precise optimization of formulation parameters, including particle size distribution, colloidal stability, and surface properties. Without such careful engineering, a nanoform may demonstrate less effective direct antibacterial action than the original, more chemically accessible Schiff base compound, which aligns with the higher MIC values observed in this work.

### Antibiofilm assay

#### Qualitative detection of biofilm formation

**Fig. 12:**
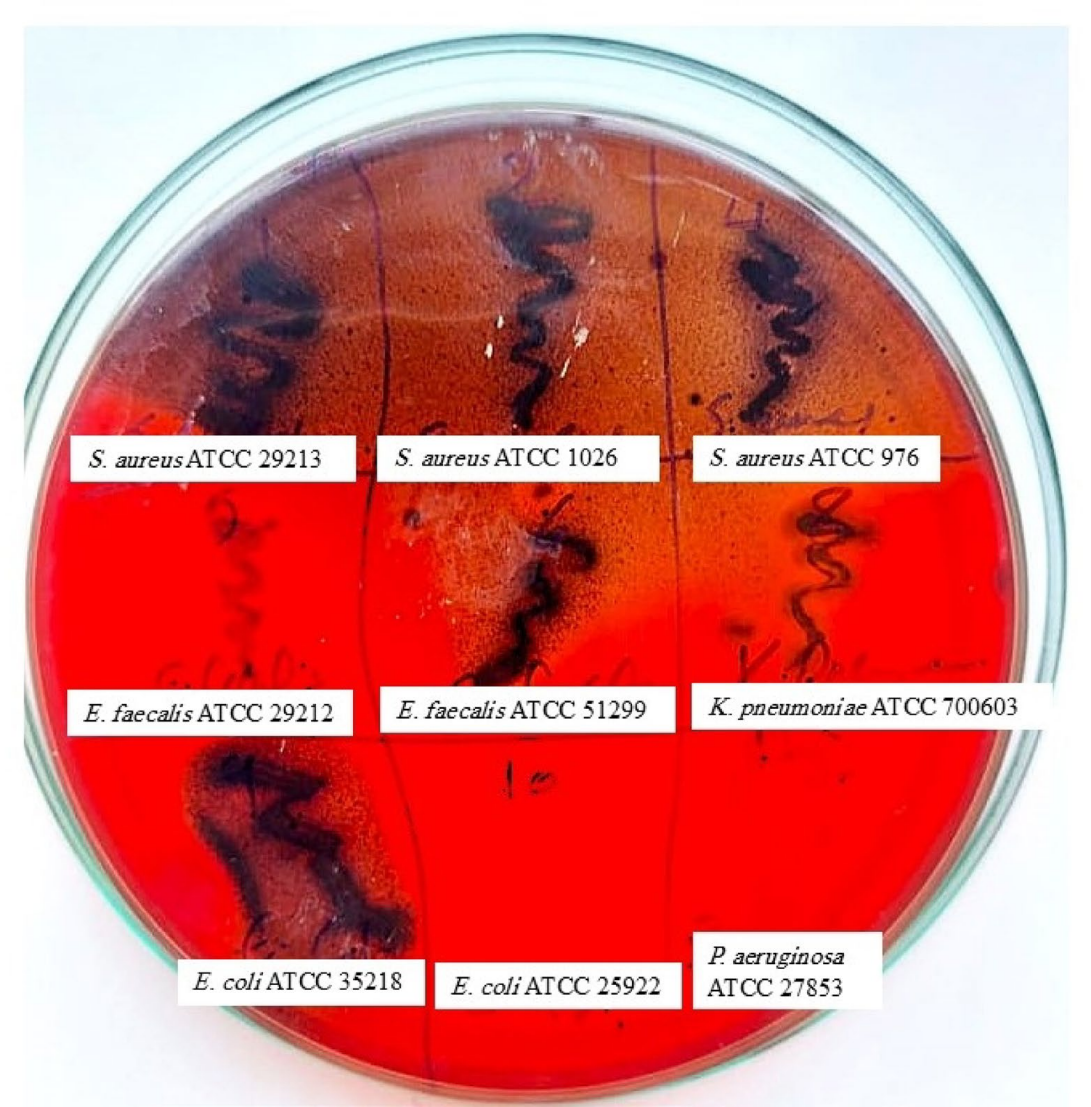
Qualitative assessment of biofilm formation on Congo Red Agar (CRA). Black colonies indicate biofilm-producing strains, while pink colonies indicate non-producers.

Biofilm formation was qualitatively assessed for all tested bacterial strains using the Congo Red Agar (CRA) method. All six bacterial strains (*S. aureus* ATCC 29213, *S. aureus* ATCC 1026, *S. aureus* ATCC 976, *E. faecalis* ATCC 51299, *K. pneumoniae* ATCC 700603, and *E. coli* ATCC 35218) were positive for biofilm production on CRA, as indicated by the characteristic colony morphologies associated with biofilm formation. Conversely, *E. faecalis* ATCC 29,212, *E. coli* ATCC 25,922, and *P. aeruginosa* ATCC 27,853 were negative, showing colony features consistent with non-biofilm producers on the same medium (Table 2; Fig. 12). The consistent Congo red binding by all *S. aureus* strains confirms their biofilm-forming capacity, validating CRA as a rapid phenotypic screening tool for clinical isolates^111^. The negative results for the reference strains *E. faecalis* ATCC 29,212, *E. coli* ATCC 25,922, and *P. aeruginosa* ATCC 27,853 are not uncommon, as CRA is known to have variable sensitivity and may fail to detect weak or atypically structured biofilms^112^. Based on these qualitative results, the strains that showed a clear positive response to CRA were selected for further quantitative analysis and inhibitory studies, ensuring that subsequent evaluation of antimicrobial compounds was conducted on confirmed biofilm producers.

**Table 2:** Results of qualitative biofilm detection using the Congo Red Agar (CRA) method.

Bacterial species	CRA phenotype (Color)	Biofilm production
*S. aureus* ATCC 29,213	Black	Positive
*S. aureus* ATCC 1026	Black	Positive
*S. aureus* ATCC 976	Black	Positive
*E. faecalis* ATCC 29,212	Pink	Negative
*E. faecalis* ATCC 51,299	Black	Positive
*K. pneumoniae* ATCC 700,603	Black	Positive
*E. coli* ATCC 35,218	Black	Positive
*E. coli* ATCC 25,922	Pink	Negative
*P. aeruginosa* ATCC 27,853	Pink	Negative

#### Quantitative assessment of biofilm biomass

The antibiofilm activity of Cs-TPA-PiP and Cs-TPA-PiP NPs was evaluated against six bacterial strains at different MIC levels (0.25×, 0.5×, and 1× MIC). Overall, both treatments exhibited concentration-dependent inhibition, with the nanoparticles demonstrating superior performance in most cases, particularly at higher concentrations. The comprehensive data for all strains and concentrations are summarized in Table 3. At sub-MIC concentrations, the tested compounds showed negative inhibition values, indicating enhanced biofilm formation. This phenomenon is known as a stress response, in which sub-inhibitory concentrations of antimicrobial agents trigger stress responses in bacteria that promote biofilm formation as a protective mechanism^113^. Against Gram-positive strains, the nanoparticles significantly outperformed the bulk compound and the positive control (Tetracycline). For instance, in *S. aureus* ATCC 976 and *E. faecalis* ATCC 51,299, the nanoform reached maximum inhibition at 1x MIC (95.83% and 95.62%, respectively), exceeding the potency of tetracycline. Interestingly, while the chitosan compound showed limited or even negative values at sub-MIC levels for some *S. aureus* strains, the nanoparticles maintained more consistent inhibitory trends. The mechanism of biofilm inhibition by chitosan and its nanoparticles involves electrostatic interactions between the positively charged chitosan and the negatively charged components of the bacterial cell surface and EPS matrix, thereby inhibiting bacterial adhesion and compromising the structural integrity of the developing biofilm. This interaction also interferes with microbial signaling and matrix production, reducing biofilm mass and bacterial adherence, particularly in both *Staphylococcus aureus* and *Pseudomonas aeruginosa*^114^.

For Gram-negative pathogens, a similar trend of nano-superiority was observed. In *K. pneumoniae* ATCC 700,603 and *E. coli* ATCC 35,218, nanoparticles at 0.5× and 1× MIC showed a marked increase in biofilm reduction compared to the chitosan compound. Specifically, at 1 x MIC, the nanoparticles achieved approximately 79.00–84.00% inhibition, significantly higher than the values recorded for both the compound and tetracycline. This potent inhibitory effect on Gram-negative pathogens can be attributed to the interference with microbial communication. It is hypothesized that biofilm formation is regulated by complex microbial communication pathways, particularly quorum sensing (QS), which controls the expression of virulence factors and the production of EPSs essential for biofilm stability. Cs-TPA-PiP and Cs-TPA-PiP NPs may significantly interfere with QS systems, thereby disrupting biofilm development even at sub-MIC levels. Previous studies have shown that in clinical isolates of *Pseudomonas aeruginosa*, chitosan nanoparticles reduced the expression of key quorum-sensing genes (*lasI* and *rhlI*), leading to near-complete inhibition of biofilm formation and motility, indicating that suppression of QS signaling is a primary mechanism of antibiofilm activity^115^. In addition to QS disruption and electrostatic interactions, chitosan nanoparticles have been shown to enhance antibiofilm efficacy by delivering quorum-quenching agents more effectively and by interacting with the biofilm matrix. Functionalized chitosan nanoparticles can adsorb onto the bacterial envelope and interfere with the transmission of signaling molecules, thereby obstructing biofilm maintenance and maturation at the molecular level. This mechanism supports the increased inhibition percentages observed with increasing nanoparticle concentration, as deeper penetration and quorum quenching prevent the establishment of stable biofilm communities^116^. Interestingly, chitosan–Schiff base compound, despite having higher MIC values against planktonic cells, Cs-TPA-PiP NPs demonstrated significantly superior antibiofilm performance. This can be attributed to the physicochemical properties of the nanoformulation. The ultra-small spherical size of the nanoparticles (15.6 nm) facilitates efficient penetration through the dense pores of the EPS matrix of established biofilms. Unlike the bulk polymer, which is hindered by steric effects, the small size of the NPs allows them to diffuse deeply into the biofilm architecture, achieving 73.00% to 95.00% biomass inhibition despite lower direct planktonic cytotoxicity.

**Table 3:** Biofilm inhibition (%) by Cs-TPA-PiP and Cs-TPA-PiP NPs at 0.25×, 0.5×, and 1× MIC.

Bacterial strain	Cs-TPA-PiP			Cs-TPA-PiP NPs			
	0.25×MIC	0.5×MIC	1×MIC	0.25×MIC	0.5×MIC	1×MIC	Positive control (Tetracycline)
*S. aureus* ATCC 29,213	−183.15^a^ ± 8.48	−53.93^b^ ± 12.15	81.27^de^ ± 2.53	−62.73^b^ ± 6.21	33.9^c^ ± 3.09	76.59^d^ ± 5.1	91.2^e^ ± 1.97
*S. aureus* ATCC 1026	−13.47^a^ ± 6.53	−9.27^ab^ ± 4.14	55.41^c^ ± 6.01	−1.10^b^ ± 4.97	50.33^c^ ± 5.66	73.95^d^ ± 1.01	47.46^c^ ± 5.97
*S. aureus* ATCC 976	40.93^b^ ± 5.16	84.31^e^ ± 1.85	86.03^e^ ± 1.95	12.99^a^ ± 4.90	75.00^d^ ± 6.54	95.83^f^ ± 3.06	62.75^c^ ± 3.47
*E. faecalis* ATCC 51,299	0.00^a^ ± 3.43	58.48^b^ ± 2.7	89.52^f^ ± 1.75	67.43^c^ ± 2.62	82.10^e^ ± 2.88	95.62 ± 0.87	75.81^d^ ± 5.43
*K. pneumoniae* ATCC 700,603	20.37^ab^ ± 6.56	16.2^a^ ± 7.13	56.94^c^ ± 5.01	17.59^a^ ± 7.13	68.06^d^ ± 6.05	79.63^e^ ± 2.89	29.63^b^ ± 7.65
*E. coli* ATCC 35,218	−16.34^b^ ± 6.3	0.65^b^ ± 11.98	60.78^c^ ± 10.38	−101.96^a^ ± 22.61	71.24^cd^ ± 7.42	84.31^d^ ± 5.19	−13.07^b^ ± 4.93

Data are expressed as mean ± standard deviation (*n* = 3). The positive control (tetracycline) was used. Different superscript letters within the same row indicate statistically significant differences (*P* < 0.05) among concentrations and compounds for each bacterial pathogen.

### Transmission electron microscopy (TEM)

**Fig. 13:**
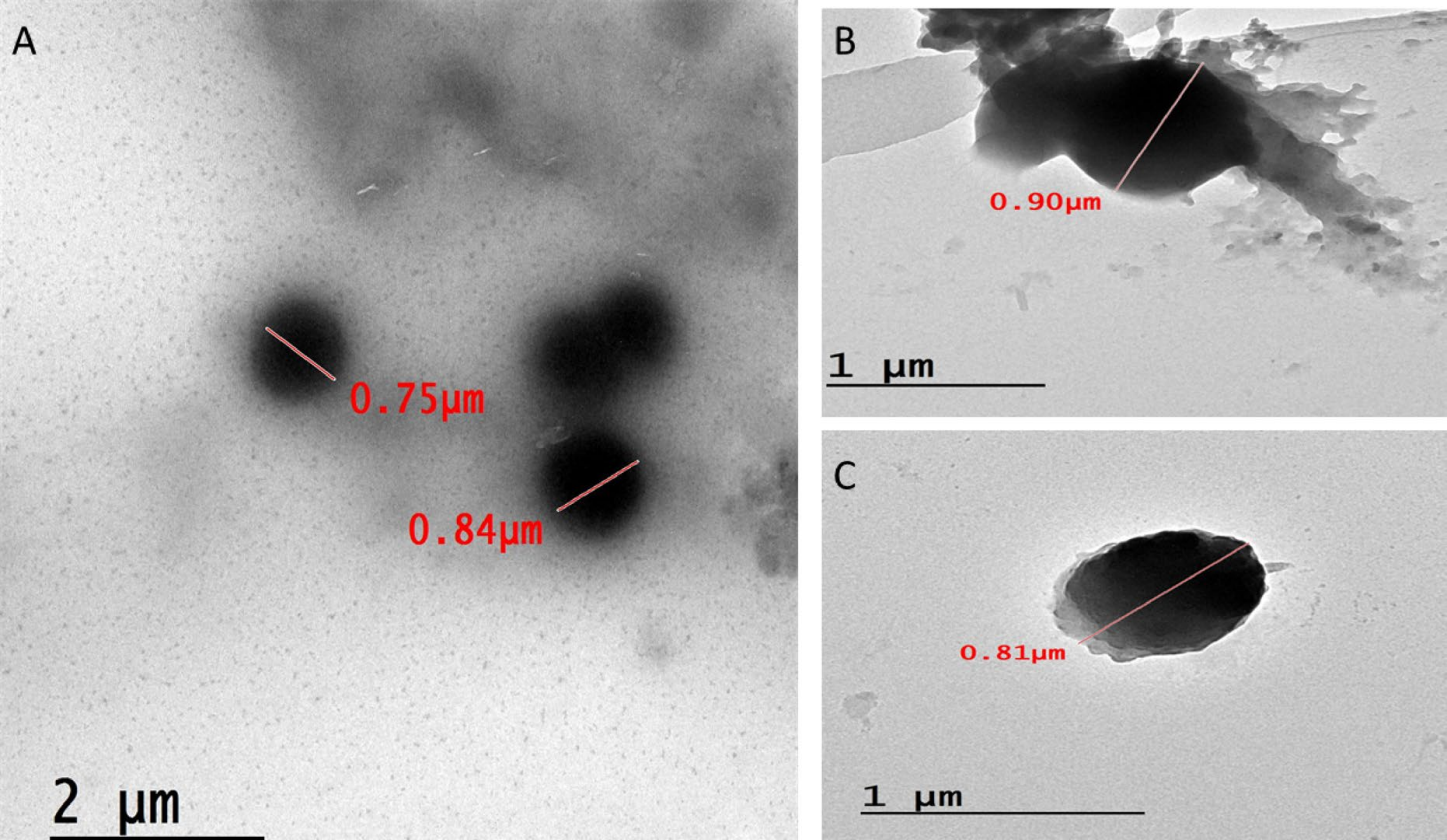
Transmission Electron Microscopy (TEM) images illustrating the morphological characteristics of *S. aureus* ATCC 1026 biofilms under various treatment conditions. (**A**) Untreated Control: Reveals normal bacterial morphology with intact cell walls, uniform small diameters, and absence of extracellular debris (Scale bar = 2 μm). (**B**) Treated with Cs-TPA-PiP: Shows a significant increase in cell diameter due to osmotic swelling and the presence of extracellular electron-dense materials indicating cell wall disruption (Scale bar = 1 μm). (**C**) Treated with Cs-TPA-PiP NPs: Reveals severe structural damage, where the bacterial cell walls appear wrinkled, shrunken, and fragmented. This indicates a total loss of membrane integrity and the leakage of intracellular constituents (Scale bar = 1 μm).

The TEM images of the untreated control samples of *S. aureus* revealed normal morphology with small and uniform diameters. The cell walls were intact, and no extracellular debris or morphological distortions were observed. The significant cell swelling observed in the treatment groups, compared to the untreated control, confirms the specific damaging effect of chitosan and its nanoparticle, as shown in Fig. 13. TEM analysis revealed a significant increase in cell diameter of treated cells by chitosan, along with the presence of extracellular electron-dense materials surrounding the bacterial cells. This morphological change can be explained by osmotic swelling, which occurs when the compound disrupts the bacterial cell wall and peptidoglycan cross-linking, impairing the wall’s ability to withstand internal turgor pressure. Consequently, water influx from the surrounding medium leads to cell expansion and eventual partial or complete rupture. Such swelling is a characteristic response of bacteria to compounds targeting cell wall integrity^117^. In addition, cell division was inhibited, resulting in abnormally enlarged cells. The bacteria treated continued to synthesize proteins and DNA, but failed to form the septum necessary for proper cytokinesis^118^.

The TEM images of *S. aureus* treated with Cs-TPA-PiP NPs revealed morphological distortions, characterized by a highly wrinkled, fragmented cell envelope. Unlike the control, the treated cells lost their circular integrity, exhibiting a corrugated surface, strongly suggesting that the nanoparticles interacted intimately with the cell wall. This interaction destabilized the membrane, leading to the observed shrunken appearance and leakage of cytoplasmic contents. The noticeable thickening and excessive wrinkling of the bacterial cell wall suggest the formation of a polymeric nano-coating layer that integrated with the cell envelope, leading to its subsequent collapse. This surface coating induces a suffocation-like mechanism, whereby the nanoparticles hinder nutrient exchange with the external medium and trap bacterial metabolites in close proximity to the cell wall. As a result, cytoplasmic leakage is accelerated, leading to membrane rupture, electron-dense debris formation, and cell lysis, as visualized in the TEM micrographs. These morphological changes confirm the potent antibacterial activity of the nanoparticles against *S. aureus*^118^. The strong affinity of nanoparticles for the bacterial cell wall enhances the local concentration of the compound at the site of action, facilitating rapid structural collapse of bacterial cells and confirming their effectiveness as antimicrobial agents^119^.

The TEM micrographs revealed that pure Cs-TPA-PiP caused severe morphological alterations and direct membrane disruption against planktonic *S. aureus* cells, indicating superior planktonic bactericidal activity. On the other hand, the chitosan-based nanoparticles, Cs-TPA-PiP NPs, demonstrated superior performance primarily within the biofilm structure, where their ultra-small size enabled deep penetration through the extracellular polymeric substance (EPS) matrix, a barrier that limits the efficacy of bulk compounds. These findings highlight two distinct mechanisms: the bulk compound acts primarily via membrane disruption, while the nanoformulation relies on EPS penetration. Hence, there is no contradiction between their respective activities.

### Molecular docking

**Fig. 14:**
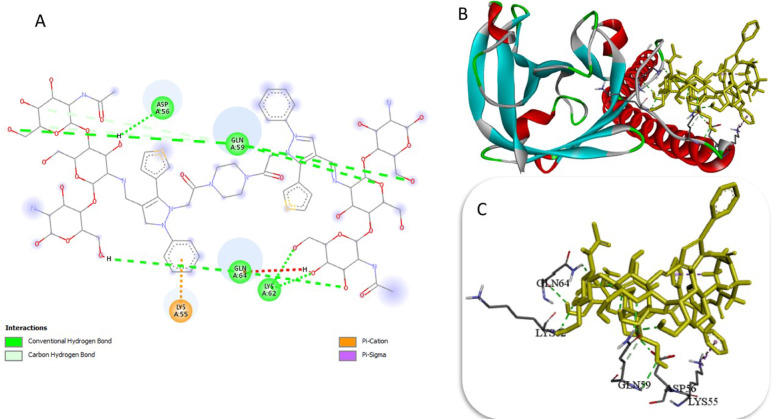
Protein-ligand complex of the tested compound chitosan–Schiff base compound Cs-TPA-PiP with the active site of Sortase A (srtA) (UniProt ID: Q2FV99). 2D molecular docking interaction of compound (**a**) and its 3D interactions within the binding pocket (**b**, **c**). The figure was generated using Discovery Studio Visualizer (version 21.1.0.20298) (BIOVIA, Dassault Systèmes, San Diego, CA, USA; https://www.3ds.com/products-services/biovia/).

**Fig. 15:**
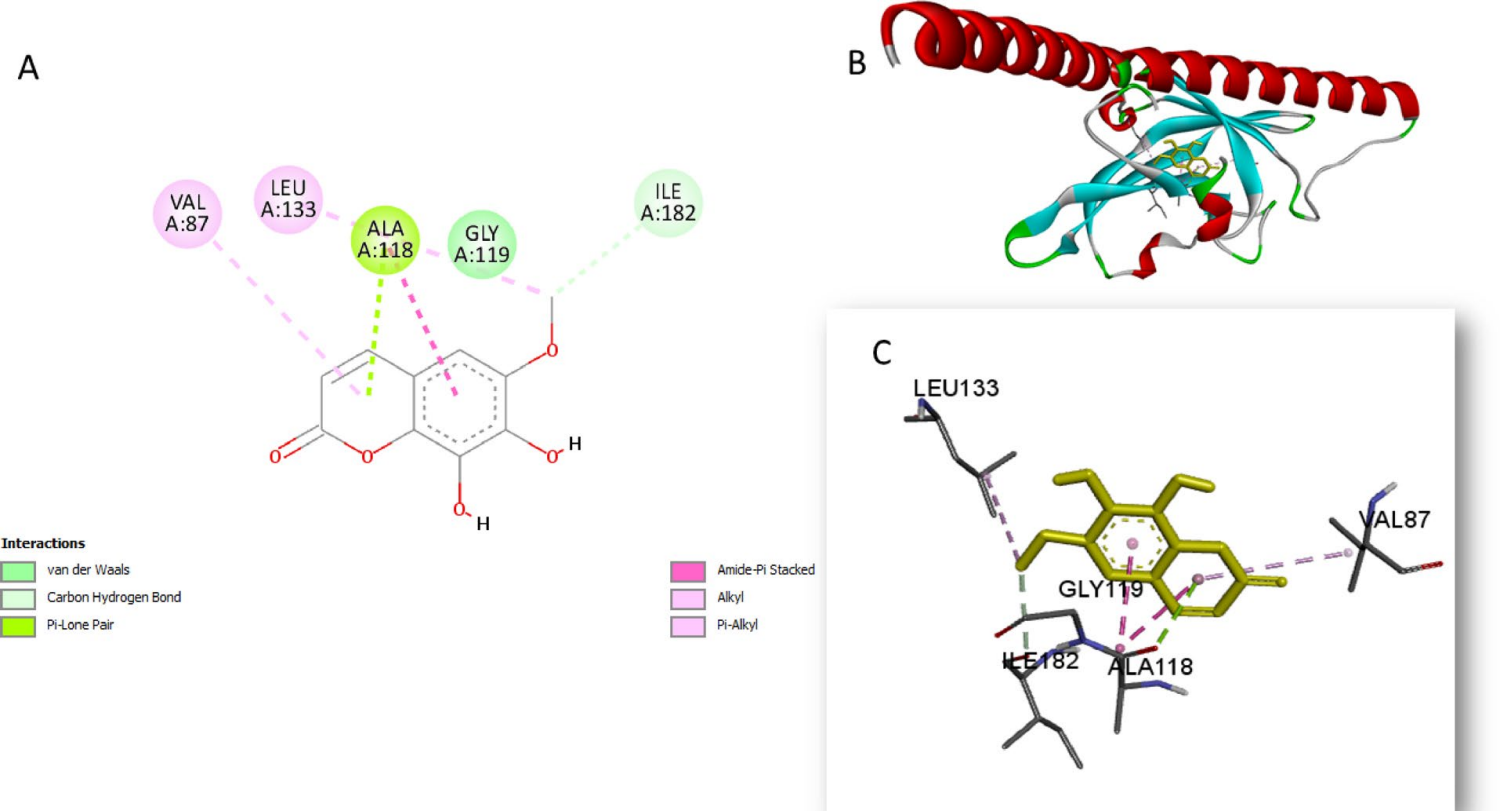
Protein-ligand complex of the reference (Fraxetin) with the active site of Sortase A (srtA) (UniProt ID: Q2FV99). 2D molecular docking interaction of compound (**a**) and its 3D interactions within the binding pocket (**b**, **c**). The figure was generated using Discovery Studio Visualizer (version 21.1.0.20298) (BIOVIA, Dassault Systèmes, San Diego, CA, USA; https://www.3ds.com/products-services/biovia/).

**Fig. 16:**
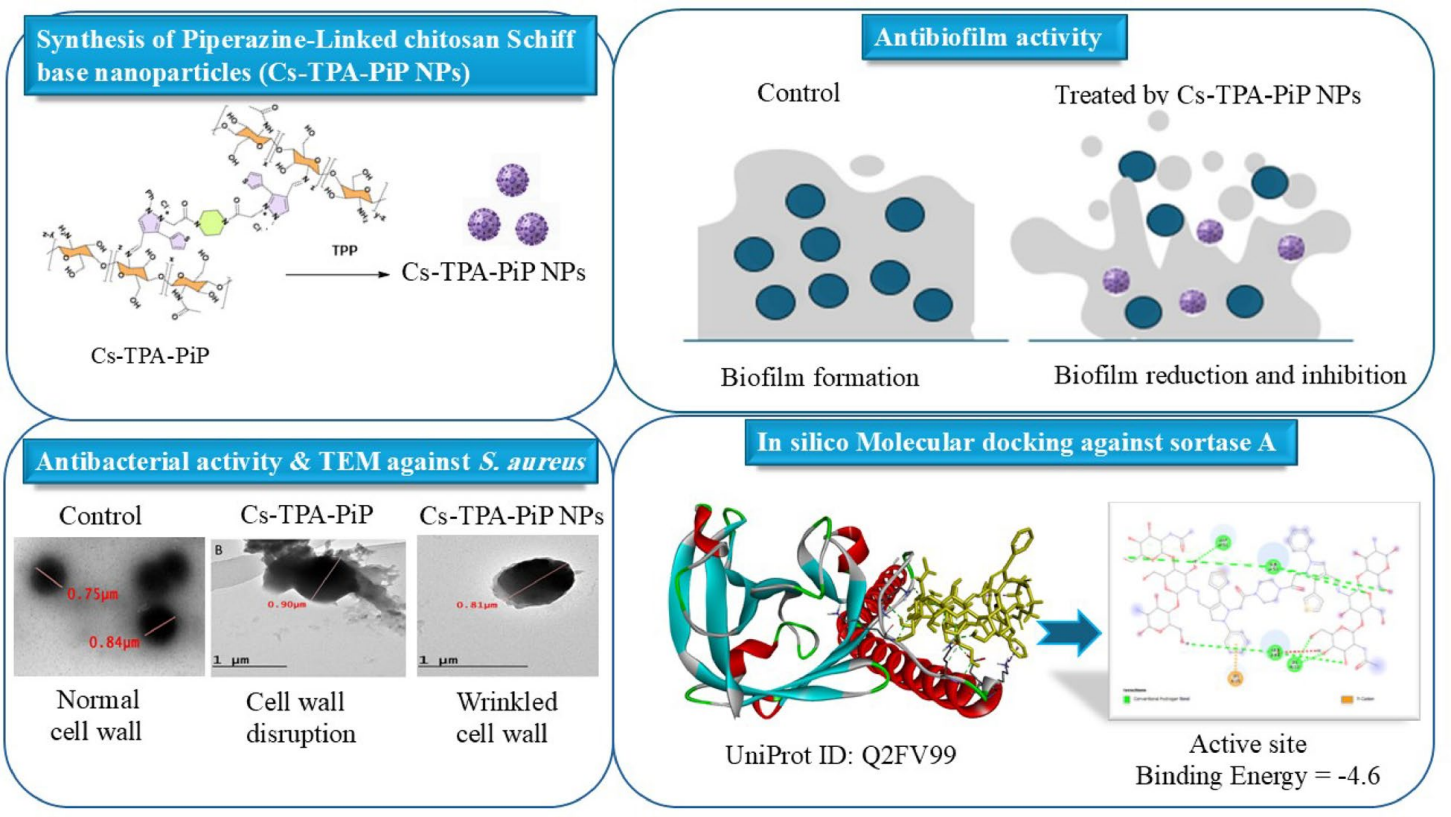
The proposed antibacterial and antibiofilm mechanisms of chitosan Schiff base nanoparticles. The figure shows the 2D and 3D binding modes, ligand structure, and TEM micrographs. The 3D molecular visualization was generated using BIOVIA Discovery Studio Visualizer (version 21.1.0.20298) (BIOVIA, Dassault Systèmes, San Diego, CA, USA; https://www.3ds.com/products-services/biovia/), and the final composite figure was assembled using Microsoft PowerPoint (Microsoft Corporation, Redmond, WA, USA).

Molecular docking was performed using AutoDock Vina version 1.5.7 to evaluate the binding affinity and interaction behavior of the synthesized compound compared with the reference drug fraxetin against Sortase A (SrtA**)** from *Staphylococcus aureus* (Table 4). Sortase A (SrtA) is a critical transpeptidase that anchors surface proteins to the cell wall of *Staphylococcus aureus*, playing an essential role in bacterial adhesion and virulence. Targeting SrtA has been widely recognized as a promising strategy for developing anti-virulence agents that reduce pathogenicity without directly inducing bacterial resistance^120^. In our study, the docking protocol revealed that the compound exhibited a binding energy of **−** 4.60 kcal/mol, whereas fraxetin showed a stronger binding affinity of **−** 6.10 kcal/mol, indicating a more stable interaction within the active site of SrtA^121^. Consistent with previous reports, fraxetin has been shown to directly reduce SrtA activity and decrease bacterial pathogenicity. Therefore, fraxetin served as an appropriate reference inhibitor for our molecular docking studies targeting Sortase A^54^. Interaction analysis showed that the synthesized compound (Cs-TPA-PiP) established multiple non-covalent interactions within the SrtA binding pocket, including Conventional hydrogen bonds, carbon–hydrogen bonds, Pi–cation interactions, and Pi–sigma interactions. These interactions involved key amino acid residues ASP56, GLN59, GLN64, LYS62, and LYS55 (Fig. 14), suggesting effective accommodation of the compound within the enzyme’s catalytic region. These residues are essential for catalysis, substrate positioning, and stabilization of the LPXTG motif during protein anchoring to the cell wall. Occupation of these residues by the compound likely interferes with enzymatic activity, which may prevent proper processing of surface proteins and could thereby reduce bacterial adhesion and virulence^122^. Binding to catalytic residues ASP56 (proton shuttle) and GLN59 (substrate geometry) may disrupt the transpeptidation network, providing a theoretical explanation for the potent SrtA inhibition^123^. Targeting key LYS residues through electrostatic and π–cation interactions may stabilize the inhibitor within the active pocket and interfere with the natural substrate, thereby impairing protein anchoring and reducing virulence^124^. In contrast, fraxetin primarily formed carbon-hydrogen, Pi-Lone pair, Amide-Pi Stacked, Alkyl, Pi-Alkyl, and van der Waals bonds with residues VAL87, ALA118, LEU133, GLY119, and ILE182 (Fig. 15), thereby contributing to its higher binding stability. The stronger binding affinity of fraxetin can be attributed to its ability to form stable hydrogen-bond networks with key residues in the active site of SrtA^125^. Although the synthesized compound exhibited lower binding affinity than fraxetin, its interaction with multiple catalytic residues via diverse bonding types, particularly π–cation and π–sigma interactions, suggests partial inhibition of SrtA activity. This moderate binding behavior correlates with the observed antibacterial activity at higher concentrations and indicates that the compound may act as a lead structure for further optimization^121^. Some chitosan Schiff derivatives dock favorably into bacterial enzyme active sites (DNA topoisomerase IV, thymidylate kinase) in molecular docking assessments, suggesting possible intracellular enzyme inhibition in addition to surface action^126^. A piperazine-based chitosan Schiff base produced marked anti-MRSA activity and showed membrane damage^127^. Quinazoline-piperazine hybrids display potent antibacterial activity and strong docking interactions with DNA gyrase active sites, raising the possibility that DNA supercoiling inhibition could contribute to their mechanism of action^128^. Piperazine conjugates bearing 1,3,4-thiadiazole/triazole fragments demonstrated antimicrobial activity, with docking results suggesting binding to bacterial enoyl-ACP reductase active sites and indicating potential inhibition of fatty acid synthesis as a proposed mechanism^129^. The proposed antibacterial and antibiofilm mechanisms of chitosan Schiff base nanoparticles are illustrated in Fig. 16.

**Table 4:** Molecular docking results and binding energies of chitosan–Schiff base compound Cs-TPA-PiP and reference controls (tetracycline) against the Sortase A (srtA) of bacterial targets.

Microbial protein target (PDB ID)	Ligand	Binding energy (ΔG, kcal/mol)	No. of interactions	Key interaction types	Key residues involved
**S**ortase A (srtA) (Q2FV99)	Compound	−4.60	11	Conventional hydrogen, Carbon hydrogen, Pi-Cation, Pi-Sigma bond	ASP56, GLN59, GLN64, LYS62, LYS55
	Fraxetin	−6.10	6	Carbon hydrogen, Pi-Lone pair, Amide-Pi Stacked, Alkyl, Pi-Alkyl, Van der Waals bond	VAL87, ALA118, LEU133, GLY119, ILE182

## Conclusion

In conclusion, this study successfully demonstrates the synthesis and comprehensive characterization of a novel piperazine-linked chitosan Schiff base (Cs-TPA-PiP) and its ionically crosslinked nanoparticle formulation (Cs-TPA-PiP NPs). The detailed characterization through FTIR and ^1^H NMR spectroscopies confirmed the successful formation of the imine linkage, the incorporation of the heterocyclic pyrazole and thiophene moieties, and the successful grafting of the piperazine core. XRD and TGA analyses further validated these structural modifications, revealing a transition from the semi-crystalline nature of pristine chitosan to a highly amorphous state and a corresponding shift in thermal stability. TEM and SEM imaging confirmed the spherical morphology and the nanometric scale of the prepared particles, which exhibited an average diameter of 15.6 nm. Both (Cs-TPA-PiP) and (Cs-TPA-PiP NPs) possess significant broad-spectrum antibacterial activity against various clinically relevant Gram-positive and Gram-negative pathogens. Although pristine chitosan Schiff base Cs-TPA-PiP exhibited lower MIC values (0.625–2.5 mg/mL) against planktonic cells compared to its nano-formulation (1–5 mg/mL), Cs-TPA-PiP NPs displayed superior efficacy in disrupting established biofilms. This enhanced antibiofilm performance, together with TEM evidence of irreversible membrane damage and molecular docking prediction of Sortase A binding affinity, suggests a multi-target mechanism of action. The observed morphological alterations, including cell wall wrinkling and fragmentation, highlight the promise of the chitosan compounds, particularly the nano-form, as potential alternatives to conventional antibiotics. These findings provide a preliminary foundation for developing chitosan-based nano therapies as effective strategies to combat persistent and resistant bacterial infections.

## Data Availability

Data availabilityAll data generated or analyzed during this study are included in this published article.
